# A Rapid One-Generation Genetic Screen in a *Drosophila* Model to Capture Rhabdomyosarcoma Effectors and Therapeutic Targets

**DOI:** 10.1534/g3.114.015818

**Published:** 2014-12-09

**Authors:** Kathleen A. Galindo, Tiana R. Endicott, Usha Avirneni-Vadlamudi, Rene L. Galindo

**Affiliations:** * Department of Pathology, University of Texas Southwestern Medical Center at Dallas, Dallas, Texas 75390-9072; † Department of Molecular Biology, University of Texas Southwestern Medical Center at Dallas, Dallas, Texas 75390-9072; ‡ Department of Pediatrics, University of Texas Southwestern Medical Center at Dallas, Dallas, Texas 75390-9072

**Keywords:** rhabdomyosarcoma, PAX7-FOXO1, PAX3-FOXO1, sarcoma, myogenesis

## Abstract

Rhabdomyosarcoma (RMS) is an aggressive childhood malignancy of neoplastic muscle-lineage precursors that fail to terminally differentiate into syncytial muscle. The most aggressive form of RMS, alveolar-RMS, is driven by misexpression of the PAX-FOXO1 oncoprotein, which is generated by recurrent chromosomal translocations that fuse either the *PAX3* or *PAX7* gene to *FOXO1*. The molecular underpinnings of PAX-FOXO1−mediated RMS pathogenesis remain unclear, however, and clinical outcomes poor. Here, we report a new approach to dissect RMS, exploiting a highly efficient *Drosophila* PAX7-FOXO1 model uniquely configured to uncover PAX-FOXO1 RMS genetic effectors in only one generation. With this system, we have performed a comprehensive deletion screen against the *Drosophila* autosomes and demonstrate that mutation of *Mef2*, a myogenesis lynchpin in both flies and mammals, dominantly suppresses PAX7-FOXO1 pathogenicity and acts as a PAX7-FOXO1 gene target. Additionally, we reveal that mutation of *mastermind*, a gene encoding a MEF2 transcriptional coactivator, similarly suppresses PAX7-FOXO1, further pointing toward MEF2 transcriptional activity as a PAX-FOXO1 underpinning. These studies show the utility of the PAX-FOXO1 *Drosophila* system as a robust one-generation (F_1_) RMS gene discovery platform and demonstrate how *Drosophila* transgenic conditional expression models can be configured for the rapid dissection of human disease.

Childhood cancer differs biologically from adult neoplasia: whereas most solid adult tumors are epithelial carcinomas, solid childhood malignancies often are mesenchymal sarcomas. Soft-tissue sarcomas account for 10% of all childhood malignancies, 50% of which are skeletal muscle-lineage rhabdomyosarcomas (RMS) (Gurney *et al.* 1999; Scheurer *et al.* 2011). Despite aggressive therapies, children with high-risk RMS suffer from a 3-yr event-free survival of 20%. Treatments for high-risk RMS have not improved for three decades, underscoring the need to elucidate the molecular underpinnings of the disease.

RMS is comprised of neoplastic myoblasts that fail to exit the cell cycle and are blocked from terminally differentiating into syncytial muscle. RMS typically is divided into two clinically distinct subgroups (Huh and Skapek 2010; Wexler *et al.* 2011): embryonal RMS and alveolar RMS (A-RMS). Embryonal RMS is a genetically heterogeneous subtype, whereas A-RMS, which is notoriously more aggressive, is uniquely driven by the PAX-FOXO1 fusion oncoprotein.

The PAX-FOXO1 transcription factor is generated by chromosomal translocations that fuse a *PAX3/7* gene (*PAX3* on chromosome 2 or *PAX7* on chromosome 1) to the 3′ end of the *FOXO1* locus on chromosome 13 (Galili *et al.* 1993; Shapiro *et al.* 1993; Davis *et al.* 1994). The encoded chimera contains intact PAX3/7 DNA-binding domains fused to the FOXO1 transcriptional activation domain (Mahajan *et al.* 2014). Because *PAX3/7* encode genetic regulators of skeletal muscle development (Lagha *et al.* 2008; Buckingham and Rigby 2014), it is postulated that genes regulated by PAX3/7 underlie A-RMS pathogenesis. Despite notable advances with mammalian PAX-FOXO1 RMS models (Keller *et al.* 2004; Naini *et al.* 2008; Nishijo *et al.* 2009; Cao *et al.* 2010), our understanding of RMS pathogenesis remains opaque, indicating the need for new genetic tools to dissect RMS pathobiology and uncover new molecular therapeutic targets.

As *Drosophila* models successfully yield critical insights into human disease, including cancer pathobiology (Gonzalez 2013), we have generated a *Drosophila* model to interrogate *in vivo* PAX-FOXO1 pathogenicity. Expression of human PAX-FOXO1 in differentiating fly muscle causes myoblast fusion defects that result in larval lethality (Galindo *et al.* 2006). Although tumorigenesis is not observed, in part due to quick lethality, PAX-FOXO1 cells act aggressively and infiltrate nonmuscle tissues. Although PAX7-FOXO1 RMS is less common and demonstrates better clinical outcomes than PAX3-FOXO1, PAX7-FOXO1 phenotypes exhibit better penetrance in flies due to slightly greater sequence identity between human PAX7 and *Drosophila* PAX3/7. Because expression of wild-type human PAX3 in flies phenocopies PAX-FOXO1, PAX3-FOXO1, PAX7-FOXO1, and wild-type PAX3/7 activity presumably overlap *in vivo* (Galindo *et al.* 2006).

PAX-FOXO1 phenotypes are susceptible to dominant genetic suppression and enhancement (Galindo *et al.* 2006; Avirneni-Vadlamudi *et al.* 2012; Crose *et al.* 2014). Thus, we have been exploiting this genetically tractable model to uncover new PAX-FOXO1 gene targets and cofactors. We have subsequently shown that genetic modifiers isolated from the *Drosophila* PAX7-FOXO1 system impact RMS oncogenesis and tumorigenesis (Avirneni-Vadlamudi *et al.* 2012; Crose *et al.* 2014). These findings establish that insights gleaned from this invertebrate model successfully uncover new RMS mechanisms, and new molecular targets for RMS therapy.

Here, we report a comprehensive deletion screen against the *Drosophila* autosomes to identify PAX-FOXO1 gene targets and effectors, as well as the methods used to configure the screen such that PAX-FOXO1 modifiers are quickly identifiable with only one genetic cross. We additionally report that mutation of *Drosophila Myocyte Enhancer Factor-2* (*D-Mef2*), a critical regulator of both fly and mammalian myogenesis, dominantly suppresses PAX7-FOXO1 lethality and acts as a PAX-FOXO1 gene target. We further find that mutation of *mastermind* (*mam*), a gene encoding a MEF2 transcriptional coactivator, similarly suppresses PAX7-FOXO1, further pointing toward MEF2 transcriptional activity as a mediator of PAX-FOXO1 pathogenicity. These studies show the utility of the PAX7-FOXO1 *Drosophila* system as a robust one-generation (F_1_) RMS gene discovery platform and demonstrate how *Drosophila* models can be configured for rapid and effective dissection of human disease.

## Materials and Methods

### Genetics

In the screen for PAX7-FOXO1 genetic modifiers, the *UAS-PAX7-FOXO1* and *Myosin Heavy Chain-Gal4* transgenes were used, and lethality assessed, as previously described (Avirneni-Vadlamudi *et al.* 2012). The Gal80-containing X-chromosome is from stock #5132 from the Bloomington *Drosophila* Stock Center. For each experimental cross, approximately three males from the master screening stock were mated to approximately five to seven wild-type, deficiency-, or gene mutation-containing females, and at least two independent crosses performed. Crosses were reared at 23°. Multiple crosses of the master screening stock to the wild-type line *w^1118^* were performed to generate large populations of F_1_ PAX7-FOXO1 male and control female siblings, from which we established a baseline percentage (22%) (SEM = 1.0%) of F_1_ PAX7-FOXO1 males expected upon routine outcrossing of the screening stock (Supporting Information, Table S1). Each time screening crosses were performed, we included new *w^1118^* control crosses to insure that PAX7-FOXO1−induced semi-lethality of F_1_ males did not significantly differ from the established baseline. All deficiency- and mutation-containing stocks were obtained from the Bloomington *Drosophila* Stock Center.

The *Drosophila* PAX7-FOXO1 microarray raw data sets have been previously described and are publically available (Avirneni-Vadlamudi *et al.* 2012).

### Embryo immunofluorescence

For *Drosophila* embryo whole-mount immunofluorescence, embryos were treated as described previously (Chen and Olson 2001), incubated in primary antibody overnight at 4° [1:1000 rabbit anti-green fluorescent protein (GFP); Molecular Probes], secondary at room temperature for 2 hr. (1:2000, Alexa-568 goat anti-rabbit; Invitrogen), and mounted in VECTASHIELD with 4′,6-diamidino-2-phenylindole. Microscopy was performed with either an LSM150-meta confocal or Zeiss Axioplan2 fluorescent microscope.

### Statistics and study approval

For the genetic screen, suppressors and enhancers were identified by crosses that showed a percent-F_1_ male population 1 SD above or below the mean, respectively. Fold change is the % F_1_ males observed for each line tested divided by baseline (22%). Unpaired 2-tailed Student’s *t*-tests were used to calculate significance. A *P* value < 0.05 was considered significant.

For the microarray studies, Data represent mean ± SEM. Significance of differences was determined by unpaired 2-tailed Student’s *t*-test. A *P* value < 0.05 was considered significant. These studies did not include human tissue, and were exempt from institutional review board approval.

## Results

### PAX7-FOXO1 drives ectopic myogenesis in *Drosophila* embryos

Because PAX molecules and myogenesis show striking evolutionary conservation between *Drosophila* and vertebrates (Halder *et al.* 1995; Xue and Noll 1996; Xue *et al.* 2001; Daczewska *et al.* 2010), we generated a genetically simple and efficient *Drosophila* PAX7-FOXO1 transgenic platform to dissect PAX-FOXO1 pathobiology. Our approach is based on the Gal4/UAS bipartite expression system (Brand and Perrimon 1993), where transgenic human PAX7-FOXO1 is expressed from the yeast *UAS* enhancer/promoter by driver lines that express the Gal4 transcriptional activator in tissue-specific patterns.

**Figure 1:**
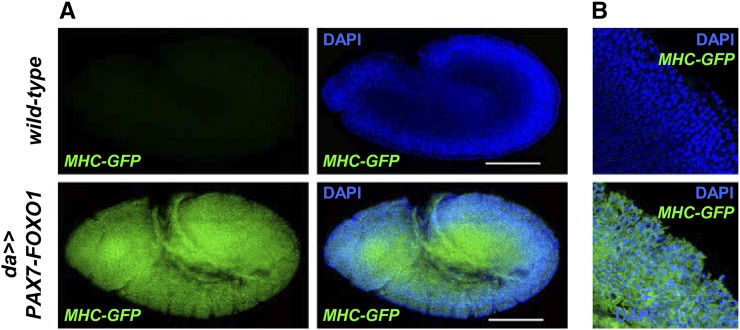
PAX7-FOXO1 drives myogenesis in *Drosophila* embryos. (A) Whole-mount *wild-type* and *daughterless-Gal4;UAS-PAX7-FOXO1* (*da>>PAX7-FOXO1*) gastrulated embryos probed for expression of green fluorescent protein (GFP) from a *Myosin Heavy Chain (MHC)-GFP* reporter transgene. Because *Drosophila* embryos initiate native expression of *MHC* at embryonic stage 13, we focused on embryos at stage 12 or younger. Diffuse expression of MHC-GFP is only detected in the *da>>PAX7-FOXO1* embryos. (B) Greater resolution images of embryo segments noted by the white bars in (A) *MHC-GFP* = GFP immunofluorescence from the *MHC-GFP* reporter; DAPI = 4′,6-diamidino-2-phenylindole nuclear staining.

Toward validating the new fly PAX-FOXO1 system, we tested whether human PAX7-FOXO1 promotes myogenesis in *Drosophila*. We used the *daughterless-Gal4* driver, which directs ubiquitous expression of *UAS*-transgenes, to express PAX7-FOXO1 during embryogenesis. We then probed for expression of a GFP-tagged *Myosin Heavy Chain* (MHC) reporter transgene, a marker specific for myogenesis and a reporter previously used in embryonic screens to successfully identify genes involved in *Drosophila* somatic muscle development and patterning (Chen and Olson 2001; Chen *et al.* 2003). *Drosophila* embryos initiate native expression of *MHC* at embryonic stage 13—thus, we focused on embryos at stage 12 or younger for ectopic *MHC* expression. We observed robust expression of *MHC-GFP* in cells of all three germ layers, including nonmyogenic cells within the ectoderm and endoderm primordia (Figure 1), findings similar to PAX3-FOXO1 misexpression in mouse embryonic primordial cells (Scuoppo *et al.* 2007). These results [as well as similar results described below (MEF2 as a PAX-FOXO gene target and putative RMS effector) (Figure 4C)] show that *Drosophila* precursors are vulnerable to the myogenic programming properties intrinsic to the PAX-FOXO1 chimera.

### A rapid, one-generation screen for PAX7-FOXO1 suppressors and enhancers

We next configured the *Drosophila* PAX-FOXO1 platform for unbiased forward genetic screening and RMS gene discovery. For these studies, we turned to an *MHC>>PAX7-FOXO1* (*MHC-Gal;UAS-PAX7-FOXO1*) genetic background to hone in on PAX7-FOXO1 pathogenicity in differentiating *Drosophila* muscle lineage cells—a setting similar to a conditional PAX3-FOXO1 tumorigenic mouse model (Keller *et al.* 2004).

**Figure 2:**
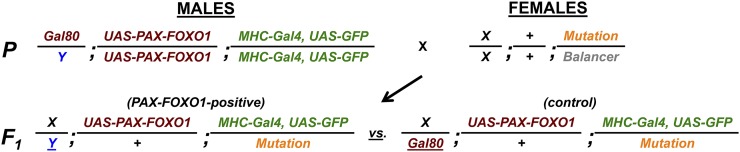
A rapid unbiased one-generation (F_1_) screen to uncover dominant PAX7-FOXO1 genetic modifiers. Incorporating an X-linked Gal80 transgenic chromosome allows for the *MHC-Gal4;UAS-PAX7-FOXO1* screen to be performed in a single generation. Using the Gal4 inhibitor, Gal80 (carried on the X-chromosome), a viable stable stock was generated that is homozygous for *UAS-PAX7-FOXO1* on the second chromosome and *Myosin Heavy Chain (MHC)-Gal4* on the third chromosome, which also contains a *UAS-GFP* transgenic reporter. With this stock, it is possible to screen against any mutant chromosome in one generation, where the number of PAX7-FOXO1-expressing F_1_ males are compared to control (Gal80-positive) female siblings. Without genetic modification, PAX7-FOXO1 expression is semilethal. Genetic suppressors rescue semilethality and thus increase the number of males in the F_1_ population, whereas enhancers decrease the percentage of F_1_ males (Table 1). In the scheme shown, the mutation tested is on the third chromosome, though an equivalent scheme is used for second chromosome mutations.

Because MHC>>PAX7-FOXO1 expression is lethal, a typical forward genetic screen would normally require a multigenerational scheme to bring the *MHC-Gal4* driver, *UAS-PAX7-FOXO1* transgene, and candidate modifiers into the same genetic background, a cumbersome and lengthy process that would need repeating for every candidate mutation-containing chromosome to be tested. To bypass this issue, we configured a stable “master” stock that would allow for candidate modifiers to be efficiently tested with a simple, one-generation (F_1_) scheme (Figure 2). To generate this master stock, we incorporated a transgenic X-chromosome that ubiquitously expresses the potent Gal4 physical inhibitor, Gal80. Because Gal80 antagonizes MHC>>Gal4, homozygous *MHC-Gal4*;*UAS-PAX7-FOXO1* animals are viable and stable. Upon outcrossing of this master stock, F_1_ female progeny inherit the Gal4-inactivating Gal80 X-chromosome (which serve as the control cohort), whereas all F_1_ male siblings express PAX7-FOXO1. Additionally, we exploited that fact that Gal4 activity is partially temperature dependent to identify a rearing temperature (23°) at which PAX7-FOXO1 phenotypes are semilethal.

Outcrossing of the screening stock to wild-type flies reared at 23° results in the PAX7-FOXO1-positive male cohort to comprise on average 22% of the F_1_ population (Table S1), significantly reduced from the rate of 50% that would otherwise be expected based on Mendelian ratios. When screening against this phenotype, PAX7-FOXO1 suppressors and enhancers are easily identified: suppressors and enhancers increase and decrease, respectively, the F_1_ percentage of males 1 SD from the mean. When compared with baseline and calculated as fold change, suppressors and enhancer show a ≥1.9-fold and ≤0.5-fold change in F_1_ male numbers, respectively (Table 1). A Student’s *t*-test is then used to confirm statistical significance for each modifier.

**Table 1:** Deficiency enhancers, suppressors, and nonmodifiers of PAX7-FOXO1

Genotype	Breakpoints	Males (P-F)	Females (Control)	Total F_1_ Adults	% F_1_ Males	Fold Change	*P* value	Submapped
*Df(3L)emc-E12*	61A;61D3	0	115	115	0%	0.00	0	No
*Df(3L)ZN47*	64C;65C	0	53	53	0%	0.00	0	No
*Df(3L)W10*	75A6-7;75C1-2	0	217	217	0%	0.00	0	No
*Df(3L)fz2*	75F10-11;76A1-5	0	77	77	0%	0.00	0	No
*Df(3R)crb-F89-4*	95D7-D11;95F15	0	225	225	0%	0.00	0	No
*Df(3R)crb87-5*	95F7;96A17-18	0	194	194	0%	0.00	0	No
*Df(2L)TW161*	38A6-B1;40A4-B1	1	112	113	1%	0.04	0	No
*Df(2R)AA21*	57B19-C1;57E1-6	1	75	76	1%	0.06	0	Yes
*Df(3R)p-XT103*	85A2;85C1-2	3	189	192	2%	0.07	< 0.0001	Yes
*Df(3L)fz-GF3b*	70C2;70D4-5	1	50	51	2%	0.09	0	Yes
*Df(2R)CX1*	49C1-4;50C23-D2	2	88	90	2%	0.10	0	Yes
*Df(2R)M60E*	60E6;60E11	5	140	145	3%	0.16	0.02	No
*Df(3L)GN24*	63F6-7;64C13-15	4	97	101	4%	0.18	< 0.0001	No
*Df(3R)23D1*	94A3-4;94D1-4	3	71	74	4%	0.18	0	No
*Df(2R)vg-C*	49B2;49E2	6	128	134	4%	0.20	0	No
*Df(3R)e-R1*	93B6-7;93D4	5	98	103	5%	0.22	0.01	No
*Df(2R)Egfr5*	57D2-8;58D1	8	140	148	5%	0.25	0	Yes
*Df(3R)ea*	88E7-13;89A1	13	175	188	7%	0.31	0.01	No
*Df(2R)BSC40*	48E1-2;48E2-10	5	66	71	7%	0.32	0.2390	No
*Df(2R)BSC161*	54B2;54B17	5	66	71	7%	0.32	0.07	No
*Df(2L)BSC32*	32A1-2;32C5-D1	7	89	96	7%	0.33	0	No
*Df(2L)TE35BC-24*	35B4-6;35F1-7	9	114	123	7%	0.33	0	Yes
*Df(2R)ED4065*	60C8;60E8	30	332	362	8%	0.38	0.01	No
*Df(2L)ast2*	21E2;22B2-3	12	128	140	9%	0.39	0.1480	Yes
*Df(2R)k10408*	54B16,54B16	20	180	200	10%	0.45	0	No
*Df(2R)BSC49*	53D9-E1;54B5-10	12	112	124	10%	0.45	0.01	Yes
*Df(3R)by10*	85D8-12;85E7-F1	19	170	189	10%	0.46	0	Yes
*Df(3R)D605*	97E2;98A5	18	152	170	11%	0.50	0.01	No
*Df(2R)M41A4*	41A;41A	22	167	189	12%	0.53	−	−
*Df(2R)Kr10*	60F1;60F5	11	80	91	12%	0.55	−	−
*Df(3R)Exel6144*	83A6;83B6	19	136	155	12%	0.56	−	−
*Df(2L)drm-P2*	23F3-4;24A1-2	16	113	129	12%	0.56	−	−
*Df(3L)66C-G28*	66B8-9;66C9-10	19	129	148	13%	0.58	−	−
*Df(2L)BSC30*	34A3;34B7-9	19	129	148	13%	0.58	−	−
*Df(3L)GN34*	63E6-9;64A8-9	42	276	318	13%	0.60	−	−
*Df(3R)Antp17*	84A5;84D9	18	106	124	15%	0.66	−	−
*Df(3R)e1025-14*	82F8-10;83A1-3	34	196	230	15%	0.67	−	−
*Df(3L)Aprt-1*	62A10-B1;62D2-5	14	80	94	15%	0.68	−	−
*Df(3R)BSC140*	96F1;96F10	17	93	110	15%	0.70	−	−
*Df(3L)ri-79c*	77B-C;77F-78A	35	190	225	16%	0.71	−	−
*Df(2R)or-BR6*	59B;59D8-E1	18	94	112	16%	0.73	−	−
*Df(2R)H3E1*	44D1-4;44F12	38	196	234	16%	0.74	−	−
*Df(2R)BSC19*	56F12-14;57A4	14	71	85	16%	0.75	−	−
*Df(3R)L127*	99B5-6;99F1	51	254	305	17%	0.76	−	−
*Df(3R)Exel6197*	95D8;95E5	22	109	131	17%	0.76	−	−
*Df(3R)B81*	99D3;3Rt	41	197	238	17%	0.78	−	−
*Df(2R)Exel7131*	50E4;50F6	29	136	165	18%	0.80	−	−
*Df(3R)Exel6202*	96C9;96E2	32	150	182	18%	0.80	−	−
*Df(3L)ME107*	77F3;78C8-9	83	387	470	18%	0.80	−	−
*Df(3L)BSC14*	67E3-7;68A2-6	21	96	117	18%	0.82	−	−
*Df(2R)en30*	48A3-4;48C6-8	22	97	119	18%	0.84	−	−
*Df(3R)Espl3*	96F1;97B1	28	123	151	19%	0.84	−	−
*Df(3R)Scr*	84A1-2;84B1-2	26	114	140	19%	0.84	−	−
*Df(3R)BSC47*	83B7-C1;83C6-D1	32	140	172	19%	0.85	−	−
*Df(2R)Jp1*	51D3-8;52F5-9	27	118	145	19%	0.85	−	−
*Df(3L)ED4978*	78D5;79A2	93	390	483	19%	0.88	−	−
*Df(2R)nap9*	42A1-2;42E6-F1	28	116	144	19%	0.88	−	−
*Df(3R)WIN11*	83E1-2;84A5	19	78	97	20%	0.89	−	−
*Df(2L)BSC41*	28A4-B1;28D3-9	33	129	162	20%	0.93	−	−
*Df(3L)brm11*	72A3;72D5	72	278	350	21%	0.94	−	−
*Df(3R)IR16*	97F1-2;98A	46	176	222	21%	0.94	−	−
*Df(2L)cl-h3*	25D2-4;26B2-5	38	145	183	21%	0.94	−	−
*Df(3L)Exel6087*	62A2;62A7	103	389	492	21%	1.0	−	−
*Df(2L)ed1*	24A2;24D4	34	126	160	21%	1.0	−	−
*Df(2R)robl-c*	54B17-C4;54C1-4	34	126	160	21%	1.0	−	−
*Df(3L)ri-XT1*	77E2-4;78A2-4	44	163	207	21%	1.0	−	−
*w^1118^ (control*, *No Df)*	N/A	314	1123	1437	22%	1.0	−	−
*Df(3L)81k19*	73A3;74F	39	134	173	23%	1.0	−	−
*Df(3R)Exel6203*	96E2;96E6	37	160	164	23%	1.0	−	−
*Df(3R)BSC137*	95A2-4;95A8-B1	43	141	184	23%	1.1	−	−
*Df(3R)Exel9012*	94E9;94E13	37	118	155	24%	1.1	−	−
*Df(2L)XE-3801*	27E2;28D1	14	44	58	24%	1.1	−	−
*Df(3R)Exel6196*	95C12;95D8	52	155	207	25%	1.1	−	−
*Df(2L)TE29Aa-11*	28E4-7;29B2-C1	39	113	152	26%	1.2	−	−
*Df(2L)FCK-20*	32D1;32F1-3	32	91	123	26%	1.2	−	−
*Df(2R)BSC44*	54B1-2;54B7-10	47	133	180	26%	1.2	−	−
*Df(2R)Px2*	60C5-6;60D9-10	39	109	148	26%	1.2	−	−
*Df(2L)ED611*	29B4;29C3	45	125	170	26%	1.2	−	−
*Df(3L)ZP1*	66A17-20;66C1-5	23	62	85	27%	1.2	−	−
*Df(3L)vin7*	68C8-11;69B4-5	47	126	173	27%	1.2	−	−
*Df(3R)M-Kx1*	86C1;87B1-5	38	99	137	28%	1.3	−	−
*Df(3L)rdgC-co2*	77A1;77D1	58	150	208	28%	1.3	−	−
*Df(3R)3450*	98E3;99A6-8	140	358	498	28%	1.3	−	−
*Df(3L)ED4782*	75F2;76A1	31	79	110	28%	1.3	−	−
*Df(2L)Prl*	32F1-3;33F1-2	35	88	123	28%	1.3	−	−
*Df(3L)eygC1*	69A4-5;69D4-6	43	107	150	29%	1.3	−	−
*Df(2R)X58-12*	58D1-2;59A	40	92	132	30%	1.4	−	−
*Df(2L)b87e25*	34B12-C1;35B10-C1	43	96	139	31%	1.4	−	−
*Df(2L)dp-79b*	22A2-3;22D5-E1	48	106	154	31%	1.4	−	−
*Df(2L)BSC36*	32D1;32D4-E1	50	106	156	32%	1.5	−	−
*Df(3R)ED5177*	83B4;83B6	50	104	154	32%	1.5	−	−
*Df(2R)PC4*	55A;55F	71	147	218	33%	1.5	−	−
*Df(3R)Exel9014*	95B1;95D1	53	109	162	33%	1.5	−	−
*Df(3L)BSC12*	69F6-70A1;70A1-2	36	74	110	33%	1.5	−	−
*Df(2R)P34*	55E2-4;56C1-11	53	108	161	33%	1.5	−	−
*Df(2L)BSC31*	23E5;23F4-5	68	138	206	33%	1.5	−	−
*Df(2R)BSC132*	45F6;46B12	43	86	129	33%	1.5	−	−
*Df(3R)Tl-P*	97A;98A1-2	90	176	266	34%	1.5	−	−
*Df(3L)pbl-X1*	65F3;66B10	41	79	120	34%	1.6	−	−
*Df(2R)BSC26*	56C4;56D6-10	48	88	136	35%	1.6	−	−
*Df(3L)st-f13*	72C1-D1;73A3-4	58	106	164	35%	1.6	−	−
*Df(2L)BSC4*	21B7-C1;21C2-3	81	142	223	36%	1.7	−	−
*Df(3L)R-G7*	62B4-7;62D5-E5	51	89	140	36%	1.7	−	−
*Df(2R)B5*	46A;46C	50	86	136	37%	1.7	−	−
*Df(2R)14H10Y-53*	54D1-2;54E5-7	58	99	157	37%	1.7	−	−
*Df(2R)w45-30n*	45A6-7;45E2-3	83	140	223	37%	1.7	−	−
*Df(2R)Exel7162*	56F11;56F16	56	94	150	37%	1.7	−	−
*Df(2L)BSC111*	28F5;29B1	58	97	155	37%	1.7	−	−
*Df(2R)ST1*	42B3-5;43E15-18	56	91	147	38%	1.7	−	−
*Df(2L)pr-A16*	37B2-12;38D2-5	47	75	122	39%	1.8	−	−
*Df(3L)XS533*	76B4;77B	42	67	109	39%	1.8	−	−
*Df(3L)BSC8*	74D3-75A1;75B2-5	53	84	137	39%	1.8	−	−
*Df(3L)vin5*	68A2-3;69A1-3	38	60	98	39%	1.8	−	−
*Df(2R)BSC39*	48C5-D1;48D5-E1	67	102	169	40%	1.8	−	−
*Df(2R)CB21*	48E;49A	56	85	141	40%	1.8	−	−
*Df(3R)3-4*	82F3-4;82F10-11	59	87	146	40%	1.8	−	−
*Df(3R)Tpl10*	83C1-2;84B1-2	41	59	100	41%	1.9	0.08	No
*Df(3R)BSC24*	85B7;85D15	61	85	146	42%	1.9	0	No
*Df(2L)JS17*	23C1-2;23E1-2	90	125	215	42%	1.9	0.02	Yes
*Df(2R)BSC22*	56D7-E3;56F9-12	67	92	159	42%	1.9	0	No
*Df(3L)BSC35*	66F1-2;67B2-3	192	262	454	42%	1.9	0.01	No
*Df(2R)BSC155*	60B8;60C4	76	103	179	42%	1.9	0.01	No
*Df(3L)BSC20*	76A7-B1;76B4-5	61	81	142	43%	2.0	0.03	No
*Df(3R)Exel6193*	94D3;94E4	58	78	136	43%	2.0	0.01	No
*Df(2L)BSC28*	23C5-D1;23E2	98	129	227	43%	2.0	0	Yes
*Df(3R)BSC42*	98B1-2;98B3-5	129	169	298	43%	2.0	0.01	No
*Df(2R)Np5*	44F12;45DE3	48	60	108	44%	2.0	0.05	No
*Df(3L)h-i22*	66D10-11;66E1-2	51	63	114	45%	2.0	0.05	No
*Df(3L)AC1*	67A2;67D11-13	47	58	105	45%	2.0	0.02	No
*Df(2L)BSC5*	26B1-2;26D1-2	77	95	172	45%	2.0	0.02	Yes
*Df(3R)Exel6195*	95A4;95B1	45	55	100	45%	2.0	0.0020	Yes
*Df(2R)vir130*	59B;59D8-E1	59	72	131	45%	2.0	0	Yes
*Df(2L)TW203*	36E-36E3;37B10	46	55	101	46%	2.1	0	No
*Df(2R)BSC18*	50D1;50D2-7	101	120	221	46%	2.1	0.02	Yes
*Df(2R)BSC29*	45D3-4;45F2-6	64	75	139	46%	2.1	0	No
*Df(2R)Exel7130*	50D4;50E4	105	120	225	47%	2.1	0.03	No
*Df(3R)mbc-R1*	95A5-7;95D6-11	99	111	210	47%	2.1	0.0050	No
*Df(2R)BSC3*	48E12-F4;49A11-B6	79	88	167	47%	2.2	0.0180	No
*Df(2R)BSC45*	54C8-D1;54E2-7	98	109	207	47%	2.2	0	No
*Df(2R)cn9*	42E;44C	59	64	123	48%	2.2	0	Yes
*Df(3L)BSC10*	69D4-5;69F5-7	62	67	129	48%	2.2	0.01	No
*Df(3L)Scf-R6*	66E1-6;66F1-6	43	45	88	49%	2.2	0	No
*Df(3L)XG5*	71C2-3;72B1-C1	67	67	134	50%	2.3	0	Yes
*Df(3L)BSC21*	79E5-F1;80A2-3	32	31	63	51%	2.3	0.28	No
*Df(2L)E110*	25F3-26A1;26D3-11	85	80	165	52%	2.3	0	Yes
*Df(3R)mbc-30*	95A5-7;95C10-11	58	52	110	53%	2.4	0.01	Yes
*Df(2L)spd[j2]*	27B2-27F2	64	55	119	54%	2.4	0.02	No
*Df(2R)X1*	46C;47A1	84	72	156	54%	2.4	0.01	Yes
*Df(2R)BSC11*	50E6-F1;51E2-4	47	36	83	57%	2.6	< 0.0001	Yes

Based on Mendalian ratios, PAX7-FOXO1−positive males would be expected to represent 50% of all F_1_ adults. At baseline (*w^1118^*), PAX7-FOXO1 expression causes semilethality, with the percentage of F_1_ PAX7-FOXO1 males reduced to an average of 22% (SEM of 1.0%) (Please see Table S1.) Enhancers and suppressors decrease and increase, respectively, survival of PAX7-FOXO1 (“P-F”) F_1_ males 1 SD from the mean (mean = 26%) (SD = 15%). “Fold Change” = % of deficiency PAX7-FOXO1 F_1_ males observed divided by baseline (22%), with enhancers and suppressors showing a fold change value of ≤ 0.5 and ≥ 1.9, respectively. *P* values were calculated for the enhancers and suppressors. Three suppressors and three enhancers did not reach statistical significance.

**Figure 3:**
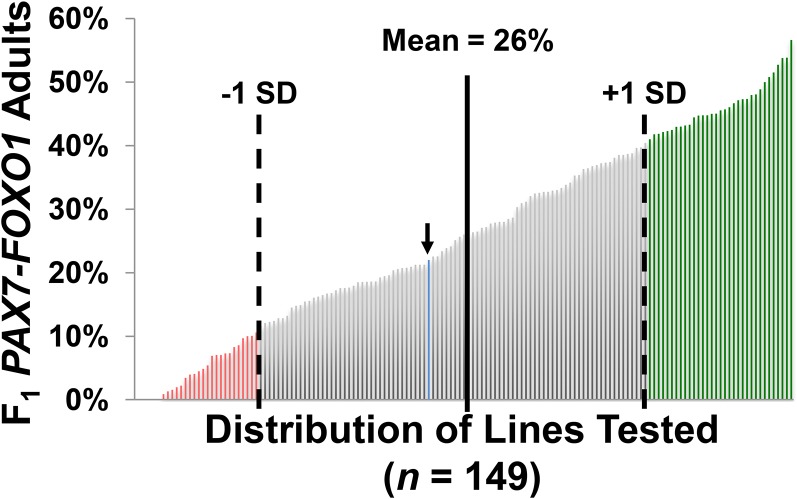
Distribution of the genetic lines tested in the PAX7-FOXO1 Screen. Shown is the plotted distribution of the tested deficiencies and the average baseline wild-type (*w^1118^*) control score (blue line, noted by the arrow) based on the percentage of F_1_ PAX7-FOXO1 males observed for each line examined. The Mean F_1_ male percentage for the screen was 26%, with a calculated SD of 15%. Suppressors (green) rank one SD above the mean, whereas enhancers (red) rank one SD below the mean.

We used a kit of minimally overlapping chromosomal deletions (*a.k.a*. “deficiencies”) (Table S1) to scan across the autosomes and identify genomic segments (or “hotspots”) that—when absent one copy—genetically modify PAX7-FOXO1 semilethality. Screening against ~95% of the *Drosophila* autosomes (~75% of the genome), we identified 33 suppressors and 28 enhancers (Table 1) (Figure 3), although three enhancers and three suppressors demonstrated *P* values above 0.05 and thus did not reach statistical significance. We next used smaller overlapping deletions to further delineate a subset of the hotspot regions, thereby significantly reducing candidate PAX7-FOXO1−interacting genes (Table 2). For the deficiency modifiers not submapped, candidate genes are provided in File S1.

**Table 2:** Submapping of PAX7-FOXO1-modifying Deficiencies

Genotype	Breakpoints	Males (P-F)	Females (Control)	Total F_1_ Adults	% F_1_ Males	Fold Change	*P* value	Comment
***Df(2R)CX1***	49C1-4;50C23-D2	2	88	90	2%	0.10	0	1
*Df(2R)Exel7123*	49D5;49E6	6	202	208	3%	0.14	0	
Candidate enhancers (49D5;49E6): *Aats-aps*, *bic*, *CG3790*, *CG3814*, *CG13319*, *CG13321*, *CG17019*, *CG30487*, *Mdr49*, *NAT1*, *Nmda1*, *Psc*, *Sans*, *sug*, *vg*								
***Df(3R)p-XT103***	85A2;85C1-2	3	189	192	2%	0.07	0	1
*Df(3R)Exel8143*	85A5;85B3	27	322	349	8%	0.36	0	
Candidate enhancers (85A5;85B3): *CG8043*, *CG8112*, *CG8116*, *CG8136*, *CG8145*, *CG8159*, *CG8202*, *CG8223*, *CG8236*, *CG9773*, *CG9801*, *CG9837*, *CG9839*, *CG11755*, *CG11760*, *CG11762*, *CG11768*, *CG13318*, *Cks85A*, *hb*, *hng2*, *Ir85a*, *M1BP*, *mRpL19*, *Pif1A*, *ranshi*, *Tcp-1eta*								
***Df(3R)by10***	85D8-12;85E7-F1	19	170	189	10%	0.46	0	1
*Df(3R)Exel6153*	85D19;85E1	4	249	253	2%	0.09	0	
Candidate enhancers (85D19;85E1): *AP-1μ*, *bocksbeutel*, *by*, *CG8199*, *CG8273*, *CG8301*, *CG8312*, *CG8319*, *CG9386*, *CG9393*, *CG9396*, *CG9399*, *CG9427*, *CG16789*, *CG16790*, *Crc*, *Kap-a3*, *MBD-like*, *mRpL47*, *mura*, *P58IPK*, *Rib1*, *RnpS1*, *Vps45*								
***Df(3L)fz-GF3b***	70C2;70D4-5	1	50	51	2%	0.09	0	2
*Df(3L)Exel6122*	70D4;70D7	109	257	366	30%	1.4	—	
Candidate enhancers (70C2-70D4): *bru-3*, *CG43184*, *CG8757*, *CG8750*, *Tsp68C*, *Hml*, *CG8745*, *dysc*, *CG13737*, *Rgl*, *CG8833*, *Glued*, *Cg32137*, *Meics*, *Nxf3*, *ssp2*, *CG13738*, *Hsc70-1*, *CG17634*, *CG17632*, *CG9040*, *26-29-p*, *CG17631*, *CG17359*, *CG8783*, *upSET*, *ptip*, *endos*, *CG6650*, *CG6661*, *Hsc70Cb*, *blue*, *CG6833*, *CG13484*, *CG32138*, *Pex1*, *breathless (FGFR)*, *CG8100*, *Fbp1*, *Sox21a*, *Sox21b*, *Dichaete*, *nan*, *nuf*, *CG32141*, *CG7768*, *CG7924*, *CG34244*, *CG7906*								
***Df(2L)E110***	25F3-26A1;26D3-11	85	80	165	52%	2.3	0	1
***Df(2L)BSC5***	26B1-2;26D1-2	77	95	172	45%	2.0	0.02	
*Df(2L)BSC184*	26B1;26B3	85	104	188	45%	2.0	0.01	
Candidate suppressors (26B1;26B3): *chickadee*, *eIF-4a*, *ifc*, *lid*, *Tsp26a*, *Gal*, *CG9098*, *H2.0*, *CG13996*, *CG9107*, *CG9109*, *mtm*, *CG9117*, *CG31643*, *ade2*, *mir-966*, *slowmo*, *CG34179*, *Cg12393*								
***Df(2R)BSC18***	50D1;50D2-7	101	120	221	46%	2.1	0.02	1
*Df(2R)50C-36*	50C19-23;50C21-D5	111	152	263	42%	1.9	0.01	
Candidate suppressors (50D1;D5): *mastermind*, *mir-4978*, *CG18371*, *Prosap*, *CG42287*, *CG42288*								
***Df(2R)BSC11***	50E6-F1;51E2-4	47	36	83	57%	2.6	< 0.0001	1
*Df(2R)L48*	50F6-F9;51B3	47	78	125	38%*	1.7*	0.04	
Candidate suppressors (50F6;51B3): *Shroom*, *CG8613*, *CG8617*, *Arc1*, *Arc2*, *Tfb1*, *CG34184*, *CG34442*, *CG4444*, *Obp50a*, *Obp50b*, *Obp50c*, *Obp50d*, *CG34185*, *Obp50e*, *CG30075*, *Dh44-R1*, *CG10104*, *CG17385*, *Sin1*, *CG17386*, *phyllopod*, *Oaz*, *Lobe*, *Cpsf160*, *Asx*, *Cpr51A*, *CG30197*, *tout-velu*, *CG30076*, *CG43919*, *LaminC*								
***Df(2L)JS17***	23C1-2;23E1-2	90	125	215	42%	1.9	0.02	2
*Df(2L)Exel7015*	23C5;23E3	59	143	202	29%	1.3	—	
Candidate suppressors (23C1;23C5): *CG8814*, *Prx6005*, *CG31950*, *betaggt-II*, *NTPase*, *lilliputian*, *Rbp9*, *Ts*, *Rrp1*, *gammaTub23C*, *CG9641*, *CG3165*, *CG9643*, *Chd1*, *Bem46*, *okra*, *CG3558*, *CG17265*, *CG17224*, *CG17264*, *alpha4GT1*, *CG3542*, *CG3605*, *CG17219,GABPI*, *CG17258*, *CG17259*, *CG17260*, *cnir*, *CG17221*, *CG17261*								
***Df(2L)BSC28***	23C5-D1;23E2	98	129	227	43%	2.0	0	2
*Df(2L)Exel7015*	23C5;23E3	59	143	202	29%	1.3	—	
Candidate suppressors (23C5;23C5): *CG17219*, *GABPI*, *CG17258*, *CG17259*, *CG17260*, *cnir*, *CG17221*, *CG17261*								
***Df(2R)cn9***	42E;44C	59	64	123	48%	2.2	0	2
*Df(2R)Exel6053*	43D3;43E9	75	138	213	35%	1.6	—	
Candidate suppressors (42E1;43D3): *CG3358*, *mim*, *CheB42b*, *CheB42c*, *Che42a*, *ppk25*, *Cyp6u1*, *CG30157*, *vimar*, *CG30156*, *CG17002*, *Tsp42E-(a-r)*, *Cg30159*, *CG30160*, *CG43646*, *CG43647*, *CG33914*, *lbm*, *pgant3*, *CG12831*, *esn*, *Cyp9b1*, *Cyp9b2*, *Spn43Aa*, *CG12828*, *prickle*, *Spn43Ab*, *Spn43Ad*, *necrotic*, *Cg11060*, *Cg33140*, *Cg30385*, *CG30384*, *Or43a*, *Ady43a*, *Gadd45*, *CG1850*, *Br140*, *Incenp*, *pawn*, *CG12164*, *Dscam1*, *costa*, *CG11107*, *Gr43a*, *CG1707*, *Eaf*, *CG11112*, *CG11113*, *CG43123*, *CG43267*, *mir-4977*, *sine oculis*, *CG11145*, *Cg11123*, *sPLA2*, *CG30503*, *kappaB-ras*, *fa2h*, *CG11127*, *p47*, *Aldh-III*, *wech*, *Coop*, *CG1620*, *dpa*, *didum*, *CG12763*, *az2*, *CG1603*, *Cg1602*, *Cg2144*, *Orc1*, *Drat*, *CG2064*, *mRpL52*, *CG12107*, *U2A*, *CG1399*, *CG30493*, *CG4096*, *CG34216*, *torso*, *CG19421*, *mir-4909*, *CG1942*, *CG1946*, *CG18812*, *CG30497*, *CG45093*, *cn*, *CanB2*, *mir-4980*, *CG12825*, *Cg12824*, *Gapdh1*, *mus205*, *Nop171*, *saxophone*, *Cg1550*, *Cg1882*, *cathD*, *CG30383*, *phr*, *phosalpha1*, *Cg18853*, *CG30382*, *CG12822*, *Atg10*, *Dgk*, *CG30377*, *CG30377*, *CG12159*, *Cul1*, *Or43b*, *Kdm4a*, *CG8791*, *CG30381*, *rnh1*, *drosha*, *CG8728*, *CG30380*, *Cg30379*, *CG14764*, *CG34430*, *CG34431*, *CG11165*, *CG30378*, *CG2906*, *CG2915*, *Sep5*, *Nito*, *CH14763*, *CG8726*, *CSN4*, *ACC*, *Nup44a*, *Dic3*, *Hey*, *CG11191*, *Odc1*, *Odc2*, *CG14762*, *mir-4981*, *Optix*, *CG12769*, *CG17977*, *lig*, *Vps28*, *slv*, *sut1*, *sut2*, *sut3*, *CG8713*, *CG8712*, *CG11210*, *Cul4*, *udd*, *Asap*, *Nup50*, *coil*, *Socs44a*, *Pbp49*, *CG42516*, *Pabp2*, *Obp44a*, *Lpin*, *kermit*, *CG8708*, *RagC-D*, *Rs1*, *CG30373*, *Gasz*, *Mlh1*, *CG14757*, *LRP1*								
***Df(2R)X1***	46C;47A1	84	72	156	54%	2.4	0.01	1
*Df(2R)BSC152*	46C1;46D7	88	124	212	42%	1.9	0.01	
*Df(2R)BSC298*	46B2;46C7	131	207	338	39%*	1.8*	0	1
*Df(2R)eve*	46C7;46C9-46C11	31	74	105	30%	1.4	—	2
Candidate genes (46B2;46C7): *CG12744*, *CG1472*, *CG1513*, *CG12923*, *CG30008*, *CG30007*, *CG1441*, *FMRFa*, *Etf-QO*, *Mef2*								
***Df(2R)vir130***	59B;59D8-E1	59	72	131	45%	2.0	0	2
*Df(2R)twi*	59C3-4;59D1-2	123	308	431	29%	1.3	—	
Candidate suppressors (59B1;59C4): *CG42260*, *CG30270*, *blw*, *CycB*, *stall*, *CG30271*, *CG42284*, *CG30274*, *CG30272*, *CG30265*, *CG12490*, *CG9825*, *CG9826*, *CG3649*, *CG13531*, *RpL23*, *inaD*, *fd59A*, *CG13532*, *PIP5K59B*, *CG3501*, *CG3499*, *asrij*, *Gmer*, *MED23*, *CG3700*, *nahoda*, *CG30187*, *Nup214*, *CG42678*, *CG3788*, *CG3800*, *CG9849*, *CG3831*, *CG42694*, *CG32834*, *CG34371*, *CG13539*, *LS2*, *CG3092*, *uip3*, *RpL22-like*, *CG12782*, *Cg13540*, *ord*, *CG3124*, *CG13541*, *Prosbeta5R1*, *HP1Lcsd*, *CG0412*, *CG30416*, *CG9861,CG30417*, *CG30413*, *CG3502*, *CG9863*, *CG34210*, *CG30409*, *Rpi*, *Cg3500*, *CG9875*, *CG34423*, *CG34424*, *vir*, *Ice1*								
***Df(3L)XG5***	71C2-3;72B1-C1	67	67	134	50%	2.3	0	
***Df(3L)brm11***	72A3;72D5	72	278	350	21%	1.0	—	
Candidate suppressors (71C2;72A3): *Best4*, *Best3*, *CG7255*, *Toll-6*, *CG33259*, *CG7804*, *Ran-like*, *CG12355*, *CG13455*, *CG7276*, *CG7275*, *Cg7272*, *CG7857*, *CG7841*, *Z600*, *gdl*, *gdl-ORF39*, *Eip71CD*, *CG13454*, *mex1*, *yellow-k*, *CG7945*, *CG33986*, *CG33985*, *CG42729*, *obst-H*, *CG42728*, *CG43248*, *CrebA*, *AGO2*, *CG7739*, *CG7427*, *dop*, *CG16979*, *mrn,CG12301*, *CG12304*, *CG7656*, *RhoGAP71E*, *CG7650*, *CG13449*, *comm3*, *CG7372*, *CG43083*, *CG43084*, *Eig71E-(a-k)*, *CG43082*, *CG7304*, *CG7579*, *pgant8*, *CG34452*, *CG34451*, *comm2*, *CG42571*, *CG42570*, *comm*, *CG6244*, *CG13445*, *fwe*, *CkIIalpha-i1*, *DCP2*, *diablo*, *CG12713*, *CG18081*, *CG15715*, *CG32150*, *mir-263b*								
***Df(3R)mbc-30***	95A5-7;95C10-11	58	52	110	53%	2.4	0.01	
***Df(3R)Exel6195***	95A4;95B1	45	55	100	45%	2.0	0.02	
***Df(3R)Exel9014***	95B1;95D1	53	109	162	33%	1.5	—	
Candidate suppressors (95A5;95B1): *CG31145*, *GILT3*, *GILT2*, *eIF-3p66*, *CG1670*, *CG18754*, *SPE*, *CG10254*, *CG10252*, *prt*, *CG31468*, *CG31148*, *CG31413*, *CG31414*, *CG10301*, *CG10300*, *nautilus (Drosophila MyoD)*, *CG10365*								
***Df(2L)ast2***	21E2;22B2-3	12	128	140	9%	0.39	0.1480**	3
*Df(2L)Exel6004*	21E4;21F1	60	80	140	43%	2.0	0.01	
Candidate enhancers (21E2;21E4): *CG2839*, *dachsous*, *Hsp60B*, *Eaat2*, *GABA-B-R3*, *CG12506*, *CG13946*, *CG13947*, *Gr21a*, *CG3544*, *Pkg21D*, *Nnf1b*, *Ddp21E2*, *Saf6*, *Pex12*, *CG15880*, *CG3867*, *clipper*, *CG3662*, *CG3862*, *dock*, *drongo*, *CG4291*, *kraken*, *CG13949*, *mir-375*, *CG13950*, *mir-375*, *aru*, *dbe*, *PNUTS*, *ninaA*, *CG15824*, *Lsp1beta*, *GluRIIC*, *CG4341*, *IA-2*, *Star*								
Candidate enhancers (21F1;22B2): *Tango14*, *CG5080*, *IntS14*, *CG14341*, *Plap*, *CG31922*, *CG5118*, *CG4887*, *CG4896*, *CG5126*, *Tgt*, *CG5001*, *Cg5139*, *CG43348*, *CG43349*, *CG5011*, *CG14342*, *CG42329*, *CG5397*, *robo3*, *a5*, *CG5440*, *CG33923*, *CG33922*, *Cdkc2*, *CG5556*, *CG5561*, *CG31924*, *CG5565*, *CG31659*, *NLaz*, *CG14346*, *leak*, *CG43401*, *CG43402*, *CG31928*, *CG33128*, *CG31926*, *CG31661*, *CG18131*, *CG7420*, *CG18132*, *halo*, *Or22a*, *CG44072*, *Or22b*, *haf*, *CG10869*, *CG31935*, *CG14352*, *RFeSP*, *chinmo*, *cpb*, *CG17660*, *mRpL48*, *frtz*, *Rim2*, *Eno*, *Rrp40*, *CG31937*, *CG17652*, *CG17646*, *CG17712*, *CG17648*, *Gr22f*, *CG17650*, *Gr22-(e-a)*, *CG31933*								
Candidate suppressors (21E4;21F1): *asteroid*, *Atg4a*, *CG4692*, *MtRNApol*, *CG14339*, *CG14340*, *Pino*, *CG4552*, *Iris*, *CG4577*, *MFS3*, *CG4749*, *Tfb4*, *Vsp29*, *capulet*								
***Df(2L)TE35BC-24***	35B4-6;35F1-7	9	114	123	7%	0.33	0	2
*Df(2L)TE35BC-7*	35B2;35B10	55	123	154	36%	1.6	—	
*Df(2L)Exel7063*	35D2;35D4	46	64	110	42%	1.9	0.03	3
Candidate enhancers (35C1;35D2): *vasa*, *vig*, *CG15270*, *CG15296*, *stc*, *CG4168*, *Sfp35C*, *CG43230*, *ZnT35C*, *dao*, *Pol32*, *l(2)35Cc*, *yuri*, *Cul3*, *UK114*, *CG15263*, *CG15260*, *ms(2)35Ci*, *CG15262*, *nht*, *esgargot*, *CG15258*, *CG44869*, *worniu*								
Candidate suppressors (35D2;35D4): *vasa*, *CG4161*, *snail*, *Tim17b2*, *lace*, *Skadu*, *CG15256*, *kek3*, *CG15255*, *Semp1*, *CG15254*, *CG15253*, *CG11865*, *Or35a*, *CG7631*, *CG18480*, *CG4578*, *CG44141*, *CG18477*, *CG18478*, *CG43923*, *CG44140*, *CG31780*, *CG1827*, *CG43924*, *CycE*								
***Df(2R)BSC49***	53D9-E1;54B5-10	12	112	124	10%	0.44	0.01	3
*Df(2R)Exel6066*	53F8;54B6	100	144	244	41%	1.9	0	
*Df(2R)BSC154*	54B2;54B7	3	93	96	3%	0.14	0.01	1
Candidate enhancers (53D9;53F8): *CG5522*, *CG15919*, *CG15615*, *CG5550*, *CG34459*, *CG34460*, *mir-8*, *Ugt37c1*, *IntS8*, *Fen1*, *Dek*, *Psi*, *Ef1beta*, *CG6426*, *CG6241*, *CG6429*, *CG6435*, *CG8910*, *CG6472*, *mir-990*, *inaC*, *Pkc53E*, *CG43788*, *CG43789*, *CG15614*, *CG43190*, *Vha16-4*, *mute*, *CG34191*, *PIG-V*, *CG6665*, *CG9010*, *Cbp53E*, *ste24c*, *CG30461*, *ste24b*, *ste24a*, *CG6796*, *NiPp1*, *CG6805*, *Ehbp1*, *CG8963*, *Dark*, *RhoGEF2*, *CG43327*, *CG43328*, *CG43371*, *CG9640*, *CG9642*, *CG9646*, *fat-spondin*, *tef*, *CG8950*, *CG6967*, *CG30460*, *Sply*, *CG6984*								
Candidate suppressors (53F8;54B2): *GstS1*, *CG30456*, *CG15611*, *Amy-p*, *CG15605*, *Cda9*, *Acp54A1*, *CG11400*, *Gbp*, *Cg11395*, *CG43103*, *CG43107*, *CG17290*, *CG17287*, *CG30458*, *CG30457*, *CG10953*, *CG10950*, *CG43237*, *muscleblind*, *CG18469*, *CG12699*, *CG43272*, *CG43108*								
Candidate enhancers (54B2;54B7): *Muscleblind*, *Sip1*, *CG6568*, *CG30101*, *Prosalpha5*, *cnk*								
***Df(2R)AA21***	57B19-C1;57E1-6	1	75	76	1%	0.06	0	2
*Df(2R)Exel6072*	57B16;57D4	30	169	199	15%	0.7	—	
*Df(2R)Exel6076*	57E1;57F3	77	89	166	46%	2.1	0.03	3
Candidate enhancers (57D4-57E1): *Rgk3*, *CG30391*, *CG30393*, *CG34023*, *MFS16*, *CG10505*, *CG30392*, *Sgf29*, *RpL29*, *CG9752*, *CG42672*, *CG9754*, *CG9485*, *CG33655*, *CG30394*, *dom*, *CG15666*, *CG9822*, *CG17974*, *cv-2*								
Candidate suppressors (57E1;57F3): *Sdc*, *Sara*, *Fkbp14*, *TAF1c-like*, *MESK2*, *CG10494*, *CG30288*, *CG30289*, *EGFR*, *CG30286*, *CG30287*, *CG33226*, *CG30283*, *twz*, *CG30222*, *CG33225*, *CG10433*, *CG15673*								
***Df(2R)Egfr5***	57D2-8;58D1	8	140	148	5%	0.25	0	3
*Df(2R)Exel6076*	57E1;57F3	77	89	166	46%	2.1	0.03	
Candidate enhancers (57D2;57E1): *CG15661*, *ASPP*, *Rgk3*, *CG30391*, *CG30393*, *CG34203*, *MFS16*, *CG10505*, *CG30392*, *Sgf29*, *RpL29*, *CG9752*, *CG42672*, *CG957*, *CG9485*, *CG33655*, *CG30394*, *domino*, *CG15666*, *CG9822*, *CG17974*, *cv-2*								
Candidate suppressors (57E1; 57F3): *CG10795*, *EfSec*, *Acox57D-p*, *Acox57D-d*, *Sdc*, *Sara*, *Fkbp14*, *TAF1C-like*, *MESK2*, *CG10494*, *CG30288*, *CG30289*, *EGFR*, *CG30286*, *CG30287*, *CG33226*, *CG30283*, *CG10440*, *CG30222*, *CG33225*, *CG10433*								

Original chromosomal deletions (*a.k.a*., Deficiencies, or Df) identified that genetically modify PAX7-FOXO1−induced male semilethality are noted in bold. Additional smaller Df’s tested to further delimit the critical modifying segments are shown directly below. Comments: 1) Df’s for which we were able to reduce the critical modifying chromosomal regions; 2) Df’s for which additional deletions tested showed no modification, indicating that the critical segments lie outside the smaller tested regions; 3) Df’s for which the smaller deletions showed opposite modifying behavior. Similar to Table 1, “Fold Change” = % of Deficiency PAX7-FOXO1 F_1_ males observed divided by the control baseline of 22%. “*” notes two smaller Df’s that, though with a fold change of slightly less than 1.9, showed a statistically significant increase in the male F_1_ population, and for this second-pass study we considered suppressors. “**” notes an original Df that did not reach statistical significance but was included in these submapping studies. “P-F” = PAX7-FOXO1.

### MEF2 as a PAX-FOXO gene target and a putative RMS effector

**Figure 4:**
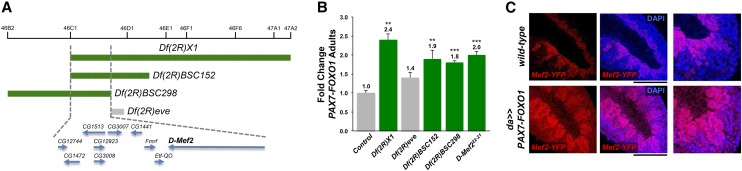
Isolation of the myogenesis benchmark gene *D-Mef2* as a PAX7-FOXO1 suppressor and gene target. (A) Smaller, overlapping chromosomal deletions reduce the PAX7-FOXO1 deletion suppressor *Df(2R)X1* to chromosomal segments 46C1-46C7, which includes *D-Mef2*, the master regulator of *Drosophila* myogenesis. (B) *D-Mef2* loss-of-function mutation dominantly suppresses PAX7-FOXO1 lethality. PAX7-FOXO1-expression is semilethal. In the presence of *Df(2R)X1*, which deletes *D-Mef2*, the population of PAX7-FOXO1−positive adults is increased 2.4-fold and is a PAX7-FOXO1 suppressor. Two smaller overlapping deletions, *Df(2R)BSC152* and *Df(2R)BSC298*, also delete *D-Mef2* and suppress PAX7-FOXO1, whereas *Df(2R)eve* neither deletes *D-Mef2* nor acts as a PAX7-FOXO1 suppressor. The *D-Mef2^22-21^* null allele (*n* = 193 F_1_ adults scored) is a strong suppressor of PAX7-FOXO1 lethality (*P* = 0.0018), confirming that *D-Mef2* genetically interacts with PAX7-FOXO1. Of note—although the *Df(2R)BSC298* deletion showed a fold change of slightly less than 1.9, the increase in PAX7-FOXO1 adults (1.8-fold) was highly significant (*P* = 0.0004), and in this test we considered a suppressor. (C) PAX7-FOXO1 drives *D-Mef2* expression. Whole-mount *wild-type* and *daughterless-Gal4;UAS-PAX7-FOXO1* (*da>>PAX7-FOXO1*) gastrulated embryos (dorsal surface upper right corner, posterior surface, lower right corner) probed for expression of yellow fluorescent protein (YFP) from a *D-Mef2-YFP* embryonic reporter transgene. In *wild-type* embryos, *D-Mef2* expression is limited to differentiating myoblasts within the mesoderm. In *da>>PAX7-FOXO1* embryos, *D-Mef2-YFP* reporter expression is seen throughout the embryo, including ectodermal and endodermal derivatives. *D-Mef2* is also detectably overexpressed in myoblasts, visible in a segmentally repeating pattern. The black lines note the posterior aspect of both embryos shown in the right-most, greater resolution images. *Mef2-YFP* = YFP immunofluorescence from the *D-Mef2-YFP* reporter; DAPI = 4′,6-diamidino-2-phenylindole nuclear staining. *P < 0.05, **P < 0.01, ***P < 0.001 *vs.* control.

We found that mutation of benchmark myogenesis genes modify PAX7-FOXO1. The *D-Mef2* gene, which operates as a linchpin and critical nodal point in fly myogenesis (Lilly *et al.* 1995), is cytogenetically located at 46C4-46C7 on chromosome 2 and is positioned within a hotspot region (46C1-47A1) initially uncovered by the *Df(2R)X1* deletion (Table 1). The hotspot region was further refined by smaller overlapping deletions to segments 46C1-46C7 (Figure 4, A and B and Table 2). Concomitantly, we found by mRNA expression profiling that *D-Mef2* is overexpressed in *PAX7-FOXO1* larval muscle (2.0-fold, *P* < 0.001, n = 3). No other gene in this region was reported as misexpressed 2.0-fold or more with statistical significance. Thus, we hypothesized that heterozygous deletion of the *D-Mef2* locus might account for *Df(2R)X1*-mediated PAX7-FOXO1 suppression, and that *D-Mef2* might act as a PAX7-FOXO1 target gene. We tested the *D-Mef2*−null mutation (Bour *et al.* 1995), *D-Mef2^22-21^*, which showed that heterozygous loss of *D-Mef2* suppresses PAX7-FOXO1 (2.0-fold increase in PAX7-FOXO1 F_1_ males) (Figure 4B). Of note, these findings do not eliminate the possibility, however, that other genes in this region might also independently interact with PAX7-FOXO1.

Because *D-Mef2* mutation suppresses PAX7-FOXO1 and *D-Mef2* is overexpressed by PAX7-FOXO1 in our microarray analysis, we investigated whether *D-Mef2* acts as a downstream PAX-FOXO1 target. We used the *daughterless-Gal4* driver to ubiquitously express PAX-FOXO1 and then probed for expression of a YFP-tagged embryonic *D-Mef2* reporter (Cripps *et al.* 1998, 1999). We found diffuse misexpression of the *D-Mef2* reporter (Figure 4C) in ectoderm and endoderm derivatives. Additionally, *D-Mef2* reporter overexpression was detected in mesodermal-derived myoblasts, visible in a segmentally repeating pattern. These studies corroborate our aforementioned *MHC-GFP* reporter expression studies, affirming that human PAX-FOXO1 promotes myogenic fate-specification in *Drosophila*. These studies further show that *D-Mef2* acts as a PAX7-FOXO1 downstream target gene (direct or indirect) and PAX-FOXO1 genetic effector *in vivo*.

**Figure 5:**
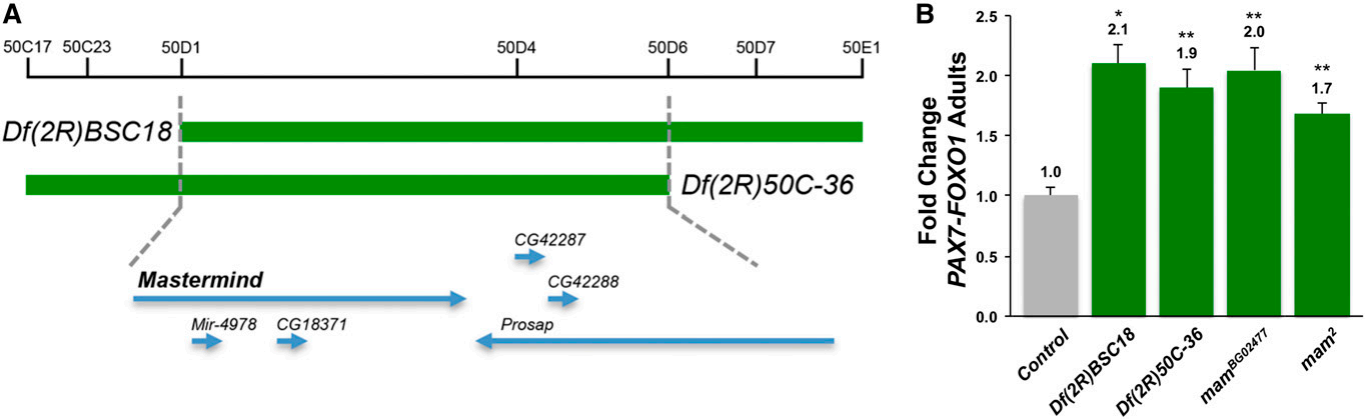
Mutation of *mastermind*, which encodes a MEF2 transcriptional cofactor, is a dominant PAX7-FOXO1 suppressor. (A) Overlapping chromosomal deletions identify a small genomic region, 50D1-50D5, as a PAX7-FOXO1−suppressing hotspot. (B) *mastermind* loss-of-function mutation dominantly suppresses PAX7-FOXO1 lethality. *Df(2R)BSC18* was isolated in our original screen as a PAX7-FOXO1 suppressor (Table 1), which deletes *mastermind* (*mam*). The overlapping deletion, *Df(2R)50C-36* also suppresses PAX7-FOXO1 (Table 2). Two well-characterized, strong loss-of-function *mam* alleles, *mam^BG02477^* (*n* = 89 F_1_ adults scored) (*P* = 0.0044) and *mam^2^* (*n* = 112 F_1_ adults scored) (*P* = 0.0043) suppress PAX7-FOXO1. Of note−although the *mam^2^* allele showed a fold change of slightly less than 1.9, the increase in PAX7-FOXO1 adults (1.7-fold) was highly significant, and in this test we scored as a suppressor. *P < 0.05, **P < 0.01, ***P < 0.001 *vs.* control.

We next interrogated the 50D1-50D5 hotspot suppressor, which contains only five genes (Table 2 and Figure 5A), one of which is *mam*. *In vivo* studies in mammalian models have shown that the *mam* ortholog *Mastermind-Like 1* (*Maml1*) encodes a transcriptional cofactor that physically interacts with Mef2 to augment Mef2-dependent promyogenic signaling (Shen *et al.* 2006; Potthoff and Olson 2007). Similar to *D-Mef2*, *mam* loss-of-function mutation dominantly suppressed PAX7-FOXO1−induced lethality (Figure 5B). Of note, *mam* expression levels were not detectably altered in our PAX7-FOXO1 microarray studies, compatible with mam’s role as a cofactor *vs.* myogenesis gene target. Taken together, these *Drosophila* studies highlight a putative PAX-FOXO1→MEF2→RMS pathogenic axis, while also demonstrating that the one-generation (F_1_) genetic screen quickly uncovers dominant PAX-FOXO1 modifiers/effectors in an unbiased fashion.

**Figure 6:**
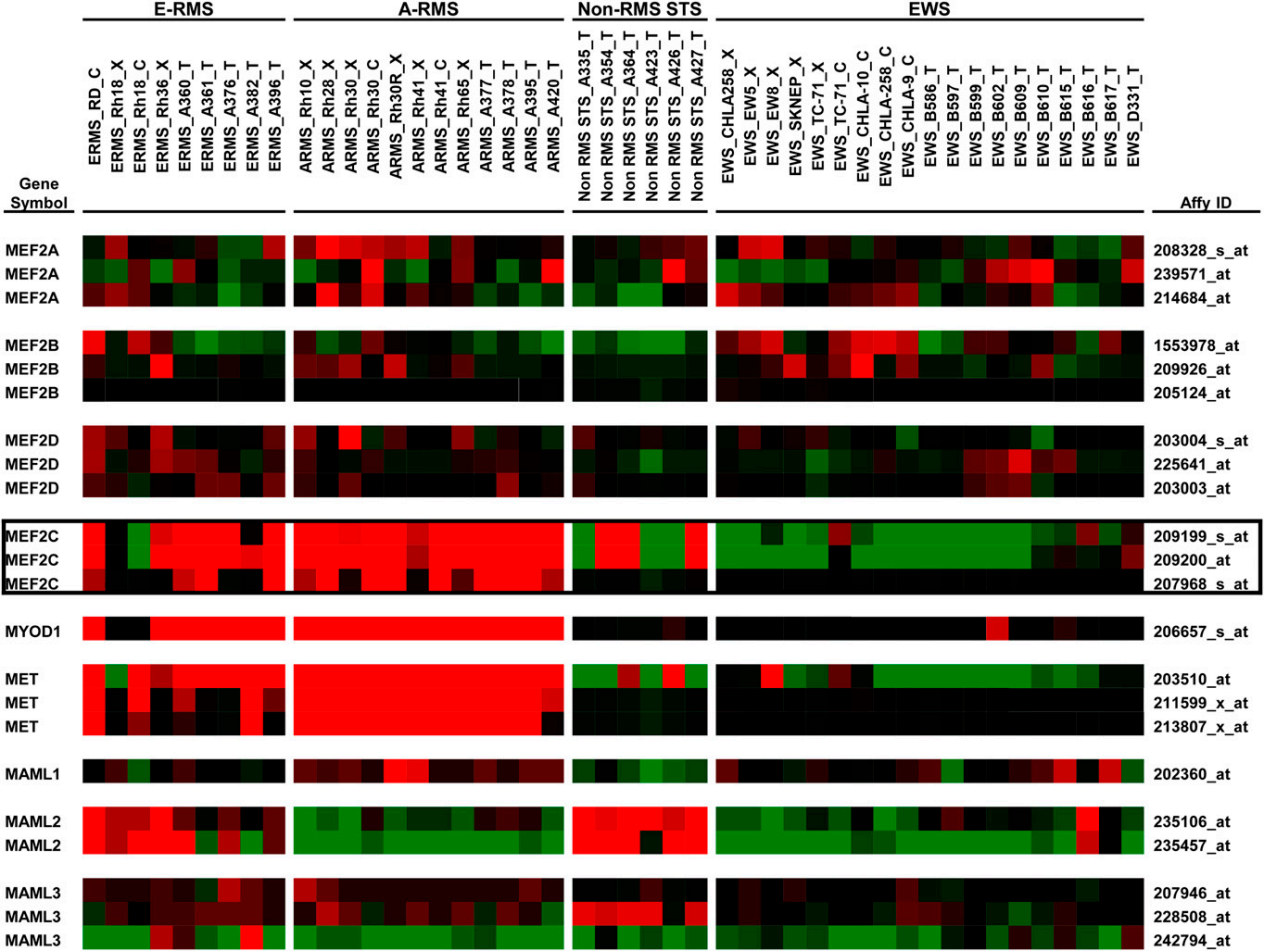
*MEF2C* is overexpressed in Rhabdomyosarcoma. Shown are expression profiles for embryonal rhabdomyosarcoma (E-RMS), alveolar rhabdomyosarcoma (A-RMS), non-RMS soft-tissue sarcoma (Non-RMS STS), and Ewing sarcoma (EWS). Profiles are from cell lines (C), tumor xenografts (X), and primary human tumors (T). Three individual probes are shown for *MEF2A*, *-B*, *-C* (bordered in black), and *-D*. Probes are also shown for the three human *Mastermind* orthologs, *MAML1*, *-2*, and *-3*. Representative probes are shown for *MYOD1* and *MET*—genes known to be up-regulated in RMS. mRNA Expression data sets are from the Pediatric Tumor Affymetrix Database (Oncogenomics; http://home.ccr.cancer.gov/oncology/oncogenomics/).

Finally, we surveyed *MEF2* expression levels in a large collection of pediatric RMS cancer cell lines, xenograft tumors, and primary tumors using the Pediatric Tumor Affymetrix Database (http://home.ccr.cancer.gov/oncology/oncogenomics/) (Khan *et al.* 2001). Four *MEF2* orthologs (*MEF2A*, *-B*, *-C*, *-D*) are present in the mammalian genome, with *-A* and *-C* demonstrating greatest similarity to *D-Mef2*. Compared with normal tissues and non-RMS pediatric soft-tissue sarcomas, only *MEF2C* showed significant and consistent up-regulation in RMS samples (Figure 6; also shown are *MYOD* and *MET*, genes that associate with RMS), data that point toward *MEF2C* as influential in RMS. Similar to *mam* in our PAX7-FOXO1 *Drosophila* system, analysis of the three mammalian *MASETRMIND* orthologs (*MAML1*, *-2*, and *-3*) did not reveal a MAML overexpression pattern in these RMS data sets (Figure 6).

## Discussion

### The *Drosophila* PAX7-FOXO1 genetic model

Given the critical role that the PAX-FOXO1 fusion oncoprotein plays in RMS, we focused on PAX-FOXO1 as an entry-point for designing a transgenic *Drosophila* RMS-related model that would be amenable to forward genetic screening and RMS gene discovery. To bypass the issue of cumbersome multigenerational screening schemes that would normally be required, we incorporated a *Gal80* X-linked chromosomal transgene to generate a viable screening *Gal4/UAS-PAX-FOXO1* master stock that allows for the rapid identification of PAX-FOXO1 genetic modifiers in a single genetic cross.

With this platform, we have been probing for new PAX-FOXO1 pathogenesis underpinnings. Though very similar in molecular structure, PAX3-FOXO1− and PAX7-FOXO1−positive RMS demonstrate differing clinical behaviors, as PAX3-FOXO1 tumors are more common and notoriously aggressive (Kelly *et al.* 1997). Consequently, PAX3-FOXO1 is the PAX-FOXO1 fusion most commonly investigated in vertebrate models. In our *Drosophila* system, we have focused on PAX7-FOXO1, which demonstrates phenotypes that are better penetrant and experimentally tractable due to the fact that human PAX7 demonstrates slightly greater sequence identity to fly PAX3/7 than does human PAX3. Additionally, as no other animal models of PAX7-FOXO1 presently exist, the fly PAX7-FOXO1 model also conveniently serves as a complement to vertebrate PAX3-FOXO1 models.

Initially unknown was the extent to which observations from the PAX7-FOXO1 fly model would impact the clinically more aggressive PAX3-FOXO1 RMS subtype, as well as PAX-FOXO1-negative (embryonal) RMS. Notably, our previous studies have shown that genetic modifiers identified from the *Drosophila* system impact PAX3-FOXO1 RMS oncogenesis and tumorigenesis (Avirneni-Vadlamudi *et al.* 2012; Crose *et al.* 2014). Furthermore, unpublished studies (U. Avirneni-Vadlamudi and R. L. Galindo, unpublished data) are demonstrating that fly PAX7-FOXO1 genetic modifiers are similarly involved in Embryonal RMS. These findings provide marked validation for the applicability and value of this genetic fly system to human RMS.

Interestingly, though PAX7-FOXO1 induces expression of the late myogenic differentiation marker MHC, PAX-FOXO1 RMS myoblasts in culture and *in vivo* demonstrate only partial differentiation with little-to-no MHC expression. In considering this discrepancy, we first note that PAX-FOXO1 is a relatively weak driver of RMS in culture and in vivo (Keller *et al.* 2004; Naini *et al.* 2008) and requires additional/sequential genetic aberrations to induce oncogenic transformation. Thus, secondary mutations might be necessary to force the strength of RMS myoblast differentiation-arrest seen in human RMS tumors; by contrast, our PAX7-FOXO1 model differs in that the system is free of any additional background mutations. Second, previous studies have shown that expression of PAX3-FOXO1 in mouse embryonic cultured cells induces the formation of MHC-positive myocytes and myotube formation (Scuoppo *et al.* 2007), studies that are similar to those seen here in the *Drosophila* system, where the *da-Gal4/UAS-PAX7-FOXO1* expression system targets undifferentiated embryonic primordia. Uncovering of the genetic/molecular sequence of RMS pathogenesis and the cell(s) origin will shed further insight into the underlying mechanisms that account for the myoblast differentiation arrest phenotypes seen in RMS *in vivo*.

### MEF2 in myogenesis and RMS

The differentiation and fusion of myoblasts into postmitotic, syncytial muscle requires that the bHLH myogenic regulatory factors (MRFs: Myf5, Mrf4, MyoD, and Myogenin) interact with E-proteins, which drive and regulate critical aspects of myogenic fate determination (Braun and Gautel 2011). The MRFs subsequently interact with the MEF2 transcription factors that, although lacking intrinsic myogenic activity, cooperate with the MRFs to synergistically activate muscle-specific genes and the downstream myogenic terminal differentiation program (Molkentin and Olson 1996; Potthoff and Olson 2007).

Vertebrates possess four *MEF2* family member genes (*-A*, *-B*, *-C*, *-D*), which demonstrate complex overlapping spatial and temporal expression patterns in embryonic and adult tissues, with greatest expression levels seen in striated muscle and brain (Potthoff and Olson 2007). Because of genetic redundancy and overlapping expression patterns of the *MEF2* genes, interrogating individual *MEF2* gene activity in mammals has been experimentally challenging, with loss-of-function mutation studies revealing only limited insights into *MEF2* gene function in tissues in which the *MEF2* genes do not overlap/compensate. Conveniently, flies possess only one *Mef2* gene (*D-Mef2*) and have served as an excellent model system to delineate MEF2’s critical role in myogenesis (Potthoff and Olson 2007). We speculate that the lack of *Mef2* redundancy in flies provided a marked experimental advantage in isolating *D-Mef2* as a PAX7-FOXO1 effector. Similarly, the identification of *mam* was also likely facilitated by the fact that flies possess one *mam* gene, whereas mammals contain three *mam* orthologs (Saint Just Ribeiro and Wallberg 2009). Thus, we propose that the comparative lack of genetic compensation/redundancy is an attractive advantage to *Drosophila* as a disease model system.

Recent studies have made significant inroads toward dissecting *MEF2* in myogenesis *in vivo* and RMS—most specifically, *MEF2C* and *-D*. Whereas global deletion of *Mef2A* or *-D* demonstrates little to no effect on embryonic myogenesis (Potthoff and Olson 2007), skeletal muscle-specific deletion of *Mef2C* causes neonatal lethality due to defective muscle integrity and sarcomere formation (Potthoff *et al.* 2007a,b). Regarding RMS, Zhang *et al.* (2013) have found that RMS cells lack proper expression of MEF2D, and that exogenous expression of MEF2D promotes RMS cell differentiation, diminishes oncogenesis in culture, and blocks tumorigenesis in xenograft studies. Turning to adult muscle regeneration and satellite stem cells, which is likely at least one cell of origin for human RMS, Liu *et al.* (2014) have now shown that *Mef2A*, *-C*, and *-D* are essential yet function redundantly in satellite cell differentiation. Lastly, our survey of published RMS microarrays (Khan *et al.* 2001), as well as PAX-FOXO1-expressing myoblast cell lines (Avirneni-Vadlamudi *et al.* 2012), shows a consistent pattern of *MEF2C* overexpression. Given the integrated and overlapping nature of the MEF2 genes, we hypothesize a potential mechanism in which overexpression of MEF2C feeds-back upon and down-regulates *MEF2D*, thereby preventing MEF2D from driving myoblast terminal differentiation.

We suggest that further interrogation of MEF2 in RMS will open new avenues for RMS chemotherapy, which for high-risk disease has not improved for decades. For example, since MEF2 activity is tightly governed by class IIa histone deacetylases (Haberland *et al.* 2007; Potthoff and Olson 2007; Nebbioso *et al.* 2009), histone deacetylase inhibitors are now ripe for preclinical testing as new anti-RMS agents. Additionally, we have found that the MEF2 cofactor Mastermind, which interacts with MEF2C and mediates crosstalk between Notch signals during myogenic differentiation (Shen *et al.* 2006; Potthoff and Olson 2007), similarly influences PAX-FOXO1 pathogenicity in flies. Interestingly, Mastermind-specific, cell-permeable peptide inhibitors have been shown to block the progression of T-cell acute lymphoblastic leukemia in mice *in vivo* (Moellering *et al.* 2009) and thus are also new agents available for RMS preclinical testing. Further characterization of *MEF2* in RMS cell and mouse models will continue to refine both our understanding and the potential targeting of *MEF2* activity in RMS.

In conclusion, we postulate that: 1) The *Drosophila* PAX7-FOXO1 model is uniquely configured for the quick uncovering of new RMS genetic effectors with one simple genetic screening cross; 2) a putative PAX-FOXO1**→**MEF2/MASTERMIND axis underlies A-RMS; and 3) *Drosophila* conditional expression models are an efficient and powerful gene discovery platform for the rapid dissection of human disease.

## Supplementary Material

Supporting Information
