# Supplementary material for: A Rapid One-Generation Genetic Screen in a Drosophila Model to Capture Rhabdomyosarcoma Effectors and Therapeutic Targets
Source: G3 (Bethesda). 2014 Dec 9;5(2):205–17. doi: 10.1534/g3.114.015818 (PMC4321029; doi:10.1534/g3.114.015818)
Supplement: Supporting Information [file supp_5_2_205__index.html]

A Rapid One-Generation Genetic Screen in a Drosophila Model to Capture Rhabdomyosarcoma Effectors and Therapeutic Targets — Supporting Information 

# A Rapid One-Generation Genetic Screen in a *Drosophila* Model to Capture Rhabdomyosarcoma Effectors and Therapeutic Targets

## Supporting Information for Galindo *et al.*, 2015

**Files in this Data Supplement:**

- File S1 - Deficiency Enhancers and Suppressors, Candidate Genes Lists. (PDF, 168 KB)
- Table S1 - Deficiencies tested for modification of PAX7-FOXO1-semi-lethality. (.xlsx, 46 KB)
