# Supplementary material for: A Rapid One-Generation Genetic Screen in a Drosophila Model to Capture Rhabdomyosarcoma Effectors and Therapeutic Targets
Source: G3 (Bethesda). 2014 Dec 9;5(2):205–17. doi: 10.1534/g3.114.015818 (PMC4321029; doi:10.1534/g3.114.015818)
Supplement: Supporting Information [file supp_g3.114.015818_015818SI.pdf]

G3 · Genes | Genomes | Genetics

A Rapid One-Generation Genetic Screen in a *Drosophila* Model to Capture Rhabdomyosarcoma Effectors and Therapeutic Targets

Kathleen A. Galindo[1], Tiana R. Endicott[1], Usha Avirneni-Vadlamudi[1], and Rene L. Galindo[1,2,3]

[1]Departments of Pathology, [2]Molecular Biology, and Pediatrics[3], University of Texas Southwestern Medical Center at Dallas, 5323 Harry Hines Boulevard, Dallas, Texas 75390-9072, USA.

Correspondence: Rene L. Galindo, M.D., Ph.D.
Department of Pathology
University of Texas Southwestern Medical Center at Dallas
Dallas, TX. 75390-9072
Phone: 214.648.4116, Fax: 214.648.4070
E-mail: rene.galindo@utsouthwestern.edu

DOI: 10.1534/g3.114.015818

File S1

Deficiency Enhancers, Candidate Genes List:

***Df(2L)BSC32*, Breakpoints: 32A1-2;32C5-D1**

Trim9, CG6138, w-cup, CG34160, CG34161, CG7329, CG31872, CG18284, CG17097, CG17098, CG45690, CG31871, CG17104, CG17105, CG17107, CG7299, CG7296, CG7294, CG17108, CG7300, Lip1, Lip2, CG6415, CG6431, CG17118, CG6750, Dpy-30L1, Nup107, Vha16-5, CG12299, CG6729, mir-4970, CG17124, CG6495, Ubc2, CG6724, CG17127, CG31869, CG31870, Nos, CG6508, CG17134, CG6700, mir-4987, CG17140, CG17139, Porin2, porin, CG43129, Dnz1, aurB, Stam, CG12517, CG14071, CG14070, CG7309, CG14069, dpr2, CG14072, CG33129, YL-1, CG16743, abo, ATPsynG, SCAR, piwi, CG12253, CG16833, aub, lectin-33A, RpL9, Nup154, Art8, dUTPase, Samuel, Acp32CD, CG14913, CG18666

***Df(2L)TW161*, Breakpoints: 38A6-B1;40A4-B1**

sick, CG10481, TotF, Victoria, COX4, CG34051, CG42866, CG13965, CG16772, CG10680, OS9, Hf, CG43861, CG10659, sNPF, barr, lok, vls, bwa, CG10730, pr, neb, fok, CG10747, CG10721, Taf13, nesd, mRpS18B, Kua, CG13970, CG10651, CG17571, CG17570, CG12617, spir, La, RtGEF, Ugt37a1, CG16798, CG10947, CG10949, Arpc2, CG15130, CG31688, CG31683, CG31687, CG18858, Cdc23, CG2493, CG43233, mir-1, CG34007, CG44270, mir-4973, CG31677, CG15475, mir-133, mir-288, CG17472, CG31680, Sfp38D, CG17470, phr6-4, CG2608, Uhg3, CG18810, CG2611, bru, CG2614, CG31678, ik2, Cen, CG2617, Hr38, CG9316, CG9317, CG9318, Fs(2)Ket, CG9319, Ns4, CG9323, dia, cad, Pomp, vari, CG9328, CG33322, CheB38b, CheB38a, CheB38c, CG9331, CG31673, CG31674, Oseg5, Spn38F, CG31676, CG14402, twit, CG14400, CG9336, CG9338, CG31675, CG14401, CG43739, sky, Mtp, RPA2, CG9272, CG9270, CG42238, Itgbetanu, CG9265, tadr, CG33511, CG33510, CG33509, ppk13, CG9259, CG14397, CG12050, CG34136, CG9257, Nhe2, Dap160, CG9253, del, E2f2, Mpp6, CG9249, CG9248, Nbr, CG9246, CG43345, CG43346, Acon, CG31627, bur, Mcm10, Ret, CG31624, CG31988, clumsy, Atg18b, CG8679, CG8677, CG31626, Hr39, l(2)k14505, CG8671, mir-4974, Cyp6t2Psi, Mio, Gr39a, crc, dimm, Tsp39D, dtr, Gr39b, CG8665, nrv3, Lamp1, nompB, CG2201, Df31, Ac3, Cul2, CG2225, EF2, CG31619, step, CG1416, CG31612, CG11630, tsh

***Df(2R)BSC40*, Breakpoints: 48E1-2;48E2-10**

CG8888, CG13186, jeb, CG18343, SkpB, CG8378, OSCP1, Hen1, CG8878, CG8407, Prp8, mir-988, CG13177, CG34232, Oda, SmD3, mir-281-2, mir-281-1, Cct5, EndoG, CG8860, wash, CG13175, CG33964, SmF, Cyp6g1, Cyp6g2, Cyp6t3, CG8858, RpS11, Sr-CII, CG13171, CG8854

***Df(2R)vg-C*, Breakpoints: 49B2;49E2**

fra, CG33752, CG30056, CG33775, Cyp301a1, Ak6, stil, ClC-b, Sin3A, Amph, Galphaq, CG45086, CG30054, CG17760, muskelin, CG33792, spt4, CG33672, CG33671, Iswi, CG8785, CG8778, ZnT49B, CG8646, nemy, CG42708, CG8771, CG13148, CG30053, Obp49a, CG8768, wuc, CG42663, mos, Taz, mRpL18, ox, Dgkepsilon, Nacalpha, CG12374, sca, CG17580, Cyp9h1, Or49b, CG17575, antr, CG30486, CG17574, bic, CG3790, CG3814, Aats-asp, Nmda1, vg, NAT1, CG13319, sug, CG17019, CG13322, Sans, CG30487, Mdr49

***Df(2R)BSC161*, Breakpoints: 54B2;54B17**
mbl, Sip1, CG6568, CG30101, Prosalpha5, cnk, l(2)k01209, CG6550, Mtap, Bap55, Lhr, EDTP, CG18467, mthl4, mthl3, CG10764, robls54B, robl, CG14478, Tes, qkr54B, insb, CG43110, veil, NT5E-2, CG30103

***Df(2R)k10408*, Breakpoints: 54B16; 54B16**
mthl3, CG10764, robls54B, robl, CG14478, Tes, qkr54B, insb, CG43110, veil

***Df(2R)ED4065*, Breakpoints: 60C8;60E8**
slik, Rpn8, SerT, CG45068, CG45069, prom, CG42383, CG15873, Pgam5-2, CG3483, CG4563, CG13579, CG3492, CG3494, CG16837, CG13589, CG13590, Crtp, Yu, Ssl, Prosalpha4T2, CG13581, Letm1, Ir60b, Ir60c, Ir60d, Ir60f, CG4612, Brca2, Ir60e, CG13585, CG4622, CG11413, ITP, Nurf-38, CG11414, uri, Fcp1, CG3511, Start1, SIFa, CG4681, pio, CG13587, ATPsynF, CG3548, CG3565, CG3570, CG4707, CG42360, CG42361, CG4741, CG3608, CG13594, Cyp9c1, CG3640, CG4781, CG3663, Cpr60D, CG30161, ND-19, CG34214, CG4806, CG33228, Pof, Mmp1, ST6Gal, Mid1, Usp15-31, spz6, CG3880, CG12848, CG3894, GstE12, Eps-15, egg, Lcp9, Eap, Aats-tyr-m, CG3589, key, Reg-5, ETH, Orc4, CG12849, CG3611, CG42851, Dll, CG3650, Atf-2, CG16896, CG30423, CG44247, NKAIN, Ance-5, NaCP60E, Tpc2, CG9083, RpL41, pain, CG30427, CG3760, CG2811, Tina-1, CG15861, CG3770, CG2790, CG12851

***Df(2R)M60E*, Breakpoints: 60E6;60E11**
Tina-1, CG15861, CG3770, CG2790, CG12851, CG2765, CG30424, RpL19, CG3776, Phk-3, CG2736, emp, CG3829, zip

***Df(3L)emc-E12*, Breakpoints: 61A;61D3**
mthl8, CG43149, Lsp1gamma, CG13405, CG12483, Pdk1, CG6845, Dic61B, p130CAS, CG7049, Vdup1, CG13875, Mkp, CG34140, Mtch, rno, mri, Gyk, NitFhit, CG13876, CG7028, thoc7, CG16940, Kaz1-ORFB, Kaz1-ORFA, pyx, CG13877, CG33229, CG42846, CG34454, CG34453, E(bx), mthl14, DIP2, wac, Tudor-SN, mRpL17, CG34263, miple, miple2, CG32845, ttm2, RhoGEF3, fwd, CG34264, CG32344, Atac3, Ptpmeg, mthl9, mthl10, mth, CG1231, Cdc5, Roc1b, CG1233, CG13884, trh, CG13891, klar, CG34267, CG34268, CG34269, CG17180, Cypl, hipk, Ppm1, Kah, MED14, CG13893, Reg-2, CG43337, ban, Gale, CG3402, MED30, Rev1, CG17129, CG3386, ebd1, CG3344, CG32483, RabX6, Vti1, CG13894, CG13895, CkIIalpha-i3, CG13896, hng3, emc, CG13898, CG13900, CG42553, CG42554, CG13901, CG13887, CG13902, Gr61a, CG13889, CG13890, dpr20, CG12502, Usp10, CG13907

***Df(3L)GN24*, Breakpoints: 63F6-7;64C13-15**
Sc2, ida, mge, Eip63F-1

***Df(3L)ZN47*, Breakpoints: 64C;65C**
Dhc64C, Aats-leu, CG13708, CG13707, CG32237, CG32235, CG17150

***Df(3L)W10*, Breakpoints: 75A6-7;75C1-2**
CG7408, CG5506, CG16775, CG7402, Tsp74F, Prestin, CG14353, CG5290, Eip75B, CG32192, CG42393, CG44006, CG44005, CG44004, CG34253, CG13698, mRpS26, CG5147, mus304, CG7341, CG32195, CG42853, Cyp312a1, gk, CG7330, CG13699, hid, CG7320, CheA75a

***Df(3L)fz2*, Breakpoints: 75F10-11;76A1-5**
fz2, CG33647, mRpL21, rept, nes, Bet1, Max, CG9666, CG42374, CG45081, CG9629, CG14085, CG14088, CG14086, CG14089, Gbs-76A, fal, pip

***Df(3R)ea*, Breakpoints: 88E7-13;89A1**
eIF-2gamma, Su(var)3-9, Set, ATPsynO, CG14864, Zip88E, Cp190, CG4338, MRG15, l(3)neo43, Tm1, CG45218, Tm2, CG14866, CG6276, CG44040, ear, CG6236, ea, mRpL9, FK506-bp1, Sra-1, CG6218, Aats-ser, Surf4, CG31301, CG42726, CG42727, Trs33, CG5038, Atg4b, CG5044, ldlCp, Trax, CG6171, CG34404, CG14868, CG6136, Ccm3, Rbp, h-cup, CG6125, Atx2, mir-13b-1, mir-13a, mir-2c, CG6118, Act88F, AdamTS-A, CG14870, Rh6, Trissin, CG5205, CG5213, CG44014, CG44013, CG31296, AOX1, AOX2, AOX3, AOX4, CG43335, CG43336, pxb

***Df(3R)e-R1*, Breakpoints: 93B6-7;93D4**
Dhc93AB, CG12278, CG31189, CG31207, CG7079, CG17279, Mvl, Cortactin, AnxB9, r-l, dmrt93B, HHEX, RhoGAP93B, CG7044, CG5745, Sec15, rtet, Rab11, ppan, Bdbt, slmb, CG5793, Obp93a, Ice2, CG7009, Usp8, meigo, SNF4Agamma, CG10824, CG5810, Snmp1, CG5862, CG3353, Oga, CG3337, Nelf-A, e, CG5892, ETHR, Rab1, AP-2sigma, CG5919, CG3308, CG3301, SIFaR, CG17298, Hsromega

***Df(3R)23D1*, Breakpoints: 94A3-4;94D1-4**
SKIP, Gld2, mir-1010, CG7084, CG34377, CG7080, CG33721, CG13862, CG5391, CG5388, CG5386, rdhB, Sar1, PSR, Muted, CG7071, CG5382, CG5380, PyK, CG7069, CG18596, CG34149, CG43342, CG43343, CG5379, CG7059, CG13857, CG13856, CG13855, CG13850, lqfR, Nop56, mats, pinta, CG13847, CG12499, CG34288, CG34376, Rpn7, AP-2mu, CG7054, Pebp1, CG5377, Nrx-1, mir-4952, CG7048, CG5376, T-cp1, CG7045, CG7046, Octbeta1R, CG5346, CG33099, CG33093, CG5326, AdipoR, bond, CG5278, CG33110, CSN6, Dph5, CG33107, CG6937, btn, Efa6, CG31156, CG13843, EF-G2, CG31161, mRpL45, loco, wake, Gclm, CG17625, wfs1, Nup133, cd, Cyp6d4, CCAP, CG6972, CG13842, CG4907, wge, Irp-1A, CG4813, CG45049, Takl2, Dcr-1, CG6985, vret, HP1c, CG17141, CG31139, rumi, CG7029, Usp12-46, CG4725, CG4723, CG4721, CG13841, CG43091, CG43092, CG43093, CG43094, CG43095, mir-4953, CG7031, CG13840, CG4704, klg, CG6660

***Df(3R)crb-F89-4*, Breakpoints: 95D7-D11;95F15**
KrT95D, Ime4, Miro, spas, Rox8, CG5986, Atg6, CG5991, CG6000, Hsp68, SMC1, 4EHP, Syx1A, CG10694, CG18428, CG13605, Rootletin, CG13607, CG5463, Tsc1, Sec10, Npc2f, Kal1, Slimp, p38c, p38a, CG6178, Myo95E, CG6182, mRpS24, Apc2, CG5510, CG13606, CHORD, CG5515, jnj, CG6204, twin, CG17786, cav, Spps, CG13609, Spase22-23, Acp95EF, mask, CG5706, jar, Orct2, Orct, CG13611, CG6356, CG34290, CG6364, CG5715, crb, Nab2, BRWD3, CG5728, Dis3, CG6432, mir-1015, Ms, CG5746, CG6454, Golgin84, CG18528, CG5762, CG33339, CG33340, CG17784, CG13613, CG42811, CG42812, CG33341, CG13614, CG17782, CG17781, CG33342

***Df(3R)D605*, Breakpoints: 97E2;98A5**
Cpr97Ea, Cpr97Eb, CG14258, CG14259, eater, CG17189, CG6074, gb, CG5815, CG6066, CG5880, CG5882

***Df(3R)crb87-5*, Breakpoints: 95F7;96A17-18**
jar, Orct2, Orct, CG13611

Deficiency Suppressors, Candidate Genes List:

***Df(2L)spd[j2]*, Breakpoints: 27B2-27F2**
nrv2, CG17376, CG17377, CG11236, CG17375, sens-2, Rca1, l(2)k09022, Nha1, Nlg2, mir-932, CG13773, Rat1, Wee1, x16, CG32829, nop5, Hrb27C, CG43232, CG18304, Fgop2, ihog, Gas41, SA, CG13775, CG3430, Atac1, CG10399, sip2, Coprox, smt3, uif, CG43321, CG43322, ade3, Pcp, CG31908, CG3476, Rab30, Caper, milt, Mnn1, CG31907, Sem1, Nuf2, CG11289, Pvf2, Pvf3, CG4495, CG4496, CG4497, CG4502, CG13784, ico, Ndae1, CG43799, CG43800, CG13786, Wnt4, CG31909, wg, Wnt6, Wnt10

***Df(2L)TW203*, Breakpoints: 36E-36E3;37B10**
CadN2, CG43271, btv, CG5674, CG5681, Prosbeta5R2, CG5693, CG42659, CG42634, CG42635, CG31740, elfless, rdo, Arr1, ninaD, CG31741, CG15153, CG5755, CG44476, CG15152, CG5758, CG31785, kel, Socs36E, CG17681, CG15155, CG5783, CG7200, CG42750, Ptp36E, CG31802, CG31788, CG31787, CG43362, Sfp36F, CG5790, CG43354, Fas3, Acp36DE, CG34171, RpS26, ncm, bsf, Ntf-2r, let-7-C, mir-100, let-7, mir-125, CG10283, CG10176, kon, CG10178, CG10211, Pde11, CG15160, amos, CG10413, CG31789, CG10333, Atac2, CG15161, MESR3, Cyp310a1, CG43338, CG43339, Mst36Fb, Mst36Fa, CG31751, tos, msl-1, CG10336, CG10383, CG10338, CG10341, CG10376, CG10343, Jwa, Faf, Grip71, Irk3, CG10348, CG42752, CG15167, ham, CG43814, CG10570, CG42502, CG42305, CG17325, CG17324, CG17323, CG17322, CG17597, ScpX, CG10600, CG31752, CG33120, CG17321, CG10602, mRpL13, tup, mir-4946, ssp3, CG10428, Nedd8, CG10621, CG10623, Nak, Tango6, CG10639, CG10431, Swip-1, CG15168, CG15170, CG15169, CG10650, CG31792, CG31793, RpL30, robl37BC, CG15172, Side, hk, CG31800, mib2

***Df(2R)Np5*, Breakpoints: 44F12;45DE3**
babo, CG8216, CG8213

***Df(2R)BSC29*, Breakpoints: 45D3-4;45F2-6**
Non1, l(2)k10201, CG33774, wun, wun2, CG13955, prel, Pdk, ced-6, Camta, CG33758, CG33757, Wnt2, brp, CG1888, mir-14, Or45b, CG1809, Not1, CG1814, CG1868, Updo, CG12929, RpL31, clos, CG1827, Map60, CG1902, CG30338, CG30340, CG30339, GstT1, GstT2, CG12926, Mmp2

***Df(2R)BSC3*, Breakpoints: 48E12-F4;49A11-B6**
CG13170, CG43315, CG43316, CG43244, CG13168, Vha36-2, Cam, CG42700, CG8850, SIP2, CG17739, CG30203, CG30046, CG13163, CG8841, garz, CG8490, CG34021, Den1, CG8839, ana3, CG30047, CG30049, CG30043, CG33012, CG13160, CG13159, Cpr49Aa, Cpr49Ab, CG13155, CG8501, Cpr49Ac, Cpr49Ad, Or49a, CG30048, Cpr49Ae, Cpr49Af, Cpr49Ag, CG30050, CG33626, CG33627, Cpr49Ah, CG13157, CG42782, CG8834, CG8520, CG8525, CG13154, Nup54, CG43204, DUBAI, CG30051, Dyb, CG30334, Lac, dgt5, CG8545, CG8550, Dh44-R2, CG34234, fdl, s-cup, vis, achi, CG13151, CG8818, CG8569, CG33632, fra, CG33752, CG30056, CG33775, Cyp301a1, Ak6, stil, ClC-b, Sin3A

***Df(2R)Exel7130*, Breakpoints: 50D4;50E4**
Prosap, CG42287, CG42288, mir-989, Rcd1, pea, CG13018, CG13016, CG8257, O-fut1, Tango7, CG8323, CG18327, CG18324, CG8331, mRpS16, cg, CG30069, VGAT, Sox15, RpS23, mir-308, CG8468, Opa1, mir-1016, CG8485, Usp20-33

***Df(2R)BSC45*, Breakpoints: 54C8-D1;54E2-7**
Klp54D, CG43324, CG14480, Ns2, POSH, UQCR-6.4, CG42239, Rab4, Dcr-2, CG6484, CG14483, RhoGAP54D, icln, CG30105, Smurf, ND-51L1, CG43920, CG42649, CG10936, CG44403, rhi, Oxp, CCHa1-R, eIF3-S8, CG30108, CG30109, P32, Sema-1b, HPS4, CG42561, CG42562, swi2, rdgBbeta, Uhg1, CG6424, CG10934, CG10933, Snx16, CG4984, CG4975, CG34195, CG6406, CG6401, CG4996, Ir54a, sub, CG10931

***Df(2R)BSC22*, Breakpoints: 56D7-E3;56F9-12**
Spt5, RpL11, Arl6, EloC, betaTub56D, CG7744, par-1, mei-W68, TBCB, Oseg6, Rep, hpo, CG15120, CG16926, CG11007, Ir56b, Ir56c, Ir56d, sm, CG43111, CG42878, CG16716, CG42753, CG18367, mir-4983, CG43277, CG15124, CG15905, Ir56e, CG15125, mir-6-3, mir-6-2, mir-6-1, mir-5, mir-4, mir-286, mir-3, mir-309, CG11018, hrg, isopeptidase-T-3, CG15127, CG34198, CG15128, CG15126, Obp56a, Obp56b, Obp56c, Obp56d, Obp56e, Obp56f, CG8517, Or56a, Obp56g, Obp56h, Toll-7, CG44569, Obp56i, CG13872, CG42690, CG43195, CG42691, CG30447, CG10822, CG44622, CG44623, CG8654, CG16898, 18w, CG11041, CG16894, CG11044, CG44624, CG44625, CG11099, CG13869, Efhc1.2, CG11208, ppk6, CG9864, Ate1, Hsl, PCNA, plu, RpS18, CG8908, CG10444, CG11788

***Df(2R)BSC155*, Breakpoints: 60B8;60C4**
yki, CG3209, Mlp60A, CG10339, CG13564, CG16786, snama, gek, enok, CG4049, CG3253, CG3257, CG43776, CG43777, CG43775, tamo, Zfrp8, CG4065, Dat, DnaJ-60, CG42568, spag, CG3328, mRpS17, Nop60B, ocm, cN-IIIB, CG3356, CG11406, CG30419, nord, Ir60a, CG13575, CG3376, CG13577

***Df(3L)h-i22*, Breakpoints: 66D10-11;66E1-2**
Pex7", h", SrpRbeta", CG32022", CG6511", CG43965", Cp18", Cp15", Cp19", Cp16", Prm", CG13306", Fhos", CG6576", CG5804", CG13310", CG13311", CG34426", CG32023", CG34427", CG13312", CG32024", CG13308", CG13309", dally", CG32026

***Df(3L)Scf-R6*, Breakpoints: 66E1-6;66F1-6**
dally, CG32026, CG43169, Mcm7, TrpA1, mfr, Tsp66E, CG43783, orb2, GNBP3, mRpL12, CG13313, CG5660, CG5653, CG5021, CG5026, pix, Srp68, CG5644, CG13314, Galk, CG5068, CG5280, smg, CG5087, Doc3, CG5194, Doc2, Doc1, CG5144, Argk, CG4911, CG4942, Tequila, CG32032, CG13315, bol

***Df(3L)BSC35*, Breakpoints: 66F1-1;67B2-3**
CG13314, Galk, CG5068, CG5280, smg, CG5087, Doc3, CG5194, Doc2, Doc1, CG5144, Argk, CG4911, CG4942, Tequila, CG32032, CG13315, bol, Dhpr, Use1, nwk, mir-4940, Rdl, Slc45-1, CG4476, CG4483, CG4477, GluRIB, PGRP-LA, PGRP-LC, PGRP-LF, UGP, CG32040, CG32039, Pdxk, CG34456, Klp67A, CG4447, CG4452, Fdxh, Hsp67Bc, Hsp67Bb, Hsp22, CG4461, Hsp26, Hsp67Ba, Hsp23, Hsp27, CG4080

***Df(3L)AC1*, Breakpoints: 67A2;67D11-13**
Slc45-1, CG4476, CG4483, CG4477, GluRIB, PGRP-LA, PGRP-LC, PGRP-LF, UGP, CG32040, CG32039, Pdxk, CG34456, Klp67A, CG4447, CG4452, Fdxh, Hsp67Bc, Hsp67Bb, Hsp22, CG4461, Hsp26, Hsp67Ba, Hsp23, Hsp27, CG4080, eIF-4E, Cpr67B, CG4022, CG3689, CG3967, CG3982, aay, Shc, RpS17, MTF-1, CG42526, Bet3, Nf-YA, CG33926, CG3529, CG3448, ghi, phol, CG3552, CG3437, CG3434, CG44838, Uch-L5, Jarid2, pall, CG32036,

CG32037, path, CG3408, RpS9, CG33703, CG33702, CG33700, Or67a, Ir67a, CG42673, CG46121, CG3222, CG3088, CG3306, LanB2, CG3335, Or67b, CG8336, fry, CG8329, CG18179, CG18180, CNMaR, CG16717, alphaTub67C, CG6767, CG16719, Ubc4, CG6761, CG16711, SH3PX1, vsg, CG18178, CG14174, nbs, defl, Naa60, ATPsynB, CG6749, mir-4986, CG42268, CG8177, Zasp67, Ilp1, CG32052, Ilp2, Ilp3, Ilp4, CG43897, Ilp5, I-2, Cdk8, RasGAP1, CG10809, iPLA2-VIA, CG8108, CG34382, Taf2, CalpB, CG6709, CG14164, CG6707, nudE, CG34356, CG6685, Dronc, CG6674, vnc, CG42455, dpr6, CG14160, CG32053, CG32054, CG42825, CG42826, dpr10, CG8072, CG6628, ect, CG8065, CG32055, scramb1, Or67c, can, Or67d

***Df(3L)BSC10*, Breakpoints: 69D4-5;69F5-7**
mirr, SmD1, Ptp69D, CG32112, CG32109, Klc, CG10984, CG10973, CG32113, Hip1, CG32106, CG10969, CG17666, Atg1, CG42709, Sap130, CG32110, CG10754, CG42588, CG10960, CG10948, Wbp2, Ent3, CG32107, CG10943, CG14120, CG43894, CG14118, CG12520, CG32117, CG32115, CG10752, Or69a, CG10748, CG10749, CG11262, CG11263, CG14117, CG11261, MICAL-like, CG11267, mRpL20, ste14, AdenoK, RpS12, Zmynd10, CG17672, SRm160, RpS4, Syx13, CG11279, CG14115, CG34428, CG34429

***Df(3L)BSC20*, Breakpoints: 76A7-B1;76B4-5**
CG32206, ms(3)76Ba, CG33062, Chd3, CG14095, CG14096, CG32214, 825-Oak, CG12519, CG18294, CG32213, brv1, pncr009:3L, CG32212, CG9449, CG9451, CG9452

***Df(3L)BSC21*, Breakpoints: 79E5-F1;80A2-3**
CG43312, CG43331, CG43330, CG43329, mir-957, CG11404, Trxr-2, CG14459, ND-B14.5AL, CG33766, CG33767, CG33768, CG33769, CG33770, CG33771, CG33772, mir-958, CG14457, CG14455, CG14456, CG14454, CG12546, CG14453, CG14452, CG32453, CG45116, CG11370, ArfGAP3, CG32452, mael, CG14451, CG14450, CG11367, CG32454, CG11241, l(3)04053, CG7369, SPoCk, CG14448, jim

***Df(3R)Tpl10*, Breakpoints: 83C1-2;84B1-2**
plx, CG2104, cas, CG1239, CG2100, CG1236, Sym, Madm, CG2091, Sec8, CG2082, jagn, kat-60L1, CG2051, Rpn5, CG1218, CG2046, CG10979, CG11000, CG42675, Hpr1, CG2023, dgrn, glob3, CG1208, CG1213, CG14676, disp, CG34287, ECSIT, CG2017, CG34113, CG11373, Obp83a, Obp83b, Rm62, CG10280, CG11459, CG15580, Or83c, Gasp, Obp83cd, Obp83ef, Obp83g, Vha14-2, CG31559, CG1077, Osi1, NPFR, Osi24, Osi2, Osi3, Osi4, Osi5, Osi6, Osi7, Osi8, Osi9, Osi10, Osi11, Osi12, CG15597, CG15594, Osi13, Osi14, Osi15, Osi16, CG46026, CG31560, Osi17, Osi18, Osi19, Osi20, CG17917, CG10298, CG17919, SmD2, CG18048, Hr83, Pak, CG42724, CG10286, CRAT, CG42564, CG1024, CG42537, TfIIFalpha, godzilla, Dmtn, gpp, CG9727, Zif, Neurochondrin, CG15186, CG15185, dpr11, Pif2, CG1137, CG2336, CG1138, CG31482, glob2, sowi, CG15177, sunz, CG31286, CG1315, Taf1, CG1307, twr, agt, lab, Edg84A, Ccp84Ag, Ccp84Af, Ccp84Ae, Ccp84Ad, Ccp84Ac, pncr002:3R, Ccp84Ab, Ccp84Aa, pb, zen2, CG34297, zen, bcd, Ama, mir-993, Dfd, mir-10, Scr, ftz, Antp, Sodh-1, CG1979, dj, djl, CG1988, CG1105, CG1965, CG1104, CG1943, Ref1, Dpck, CG1091, alphaTub84B

***Df(3R)BSC24*, Breakpoints: 85B7;85D1**
pyd, osk, skap, CG11964

***Df(3R)Exel6193*, Breakpoints: 94D3;94E4**

klg, CG6660, Tpl94D, CG17244, Nha2, Or94a, Or94b, CG34375, CG6688, lmd, CG13833, CG13838, CG13837, CG17121, p53, Gr94a, CG17119, sav, Ublcp1, CG17111, CG13830, CG17110, CG6726, CG17109, CG6733, CG6738, Rassf, CenB1A, CG31365, CG31457, hh, unk, VhaAC39-2, CG13829, Irk1, cnc

***Df(3R)mbc-R1*, Breakpoints: 95A5-7;95D6-11**
CG31145, GILT3, GILT2, eIF-3p66, CG16710, CG18754, SPE, CG10254, CG10252, prt, CG31468, CG31148, CG31413, CG31414, CG10301, CG10300, nau, CG10232, CG10365, Rpn9, Hmgcr, RanBP3, Rpt5, Hrd3, Plip, SdhD, CG10217, Lsd-1, CG10214, CG10375, tst, CG10208, Nup98-96, mbc, CG33111, CG34355, eIF4G2, Pli, TfIIA-S, tbrd-1, sba, CG31141, Ndc1, CG13601, CG43998, CG43999, CG31142, Rpt2, CG13599, RpS19b, CG5854, Gdh, CG12268, Aats-glupro, AP-1sigma, CG31140, CG13603, CG5902, CG13604, Rab7, CG33108, LSm3, KrT95D, Ime4, Miro, spas, Rox8, CG5986, Atg6, CG5991, CG6000, Hsp68, SMC1, 4EHP

***Df(3R)BSC42*, Breakpoints: 98B1-2;98B3-5**
unc80, CG5590, CG12883, Sce, CG31055, CG12880, CG43319, Acp98AB, CG43320, ALiX, btz, CG12428, CG12877, Ets98B, CG34353, Or98P, Or98a, CG12426, mil, CG5003
